# Silent Inflammation: A Critical Narrative Review of the Relationship Between Periodontal Disease and Psychosis—The Role of Oxidative Stress and Iatrogenic Comorbidities

**DOI:** 10.3390/antiox15060679

**Published:** 2026-05-28

**Authors:** Brindusa E. Focseneanu, Roxana M. Ciobanu, Anna M. Pangica, Petru T. Ionescu, Teodora M. Pangica, Gabriela Marian, Florentina C. Biclesanu

**Affiliations:** 1 Faculty of Medicine, Titu Maiorescu University, 031593 Bucharest, Romania; gabriela.marian@prof.utm.ro; 2 Faculty of Dental Medicine, Titu Maiorescu University, 031593 Bucharest, Romania; anna-maria.pangica@prof.utm.ro (A.M.P.); tudor.ionescu@prof.utm.ro (P.T.I.); teodora.pangica@s.utm.ro (T.M.P.); florentina.biclesanu@prof.utm.ro (F.C.B.); 3 Academy of Romanian Scientists, Section of Medical Sciences, 050044 Bucharest, Romania

**Keywords:** periodontitis, schizophrenia, psychosis, oxidative stress, redox dysregulation, TXNIP–NLRP3 axis, AGEs–RAGE axis, mitochondrial dysfunction, biomarkers, integrated care

## Abstract

Extensive epidemiological evidence links psychosis (PZ)—particularly schizophrenia (SCZ)—with disproportionate periodontal destruction, suggesting shared biological vulnerability. Beyond local tissue damage, periodontitis provides a clinically accessible translational paradigm for systemic redox dysregulation, where sustained inflammatory activation coincides with measurable oxidative injury and exhaustion of antioxidant (AO) defenses across cardiometabolic and neuropsychiatric domains. In this critical narrative review, we argue that the excess periodontal burden in PZ reflects a “pathological confluence” shaped by antipsychotic-associated iatrogenic factors, rapid metabolic deterioration, and chronic oxidative distress. We appraise the thioredoxin-interacting protein (TXNIP)–NOD-like receptor (NLR) family pyrin domain-containing 3 (NLRP3) axis as a metabolic–redox sensor linking dysglycemia to periodontal inflammasome activation and downstream cytokine signaling, and address the advanced glycation end-products (AGEs)–receptor for advanced glycation end-products (RAGE) axis as a key immunometabolic redox pathway. We further discuss mitochondrial dysfunction, impaired mitophagy, and mitochondrial deoxyribonucleic acid (mtDNA) leakage as damage-associated molecular patterns (DAMPs) that can amplify systemic “silent inflammation”. Integrating evidence on periodontal pathogen–host interactions and redox-sensitive neuroimmune pathways (including NADPH oxidase 4 (NOX4)-linked microglial activation), we propose periodontitis as a plausible upstream amplifier that may exacerbate vascular dysfunction and compromise blood–brain barrier (BBB) integrity. Finally, we outline clinically measurable biomarker readouts to operationalize redox-informed integrated care and highlight the need for pragmatic trials targeting clinically meaningful endpoints to improve somatic longevity in PZ-spectrum populations. We acknowledge that current human evidence is largely associative and that the proposed mechanistic links remain hypothesis generating.

## 1. Introduction: The Dual Burden of Inflammation

### 1.1. Global Health Urgency and Systemic Susceptibility in Psychosis (PZ)

PZ and schizophrenia (SCZ) pose significant public health challenges, and their associated physical health needs—including oral health disparities—can substantially affect quality of life [1,2]. Individuals with SCZ experience a substantial mortality gap of ~15–20 years relative to the general population, which is largely attributable to preventable/modifiable physical comorbidities and health-risk factors [3].

Recent longitudinal findings from the Chinese First-Episode Schizophrenia Trial (CNFEST) indicate that metabolic decline is not uniform; a high-risk low baseline body mass index (BMI) with rapid increase (LBRI) subgroup shows notable weight gain (+3.5 kg/m^2^) within the initial three months of commencing second-generation antipsychotics (SGAs), such as olanzapine [4,5].

This excessive somatic burden is underpinned by cardiometabolic vulnerability accompanied by chronic low-grade systemic inflammation and immune–metabolic dysregulation [2,6], with high rates of metabolic syndrome (MetS) reported in SCZ [7]. Traditionally, “systemic spillover” from the periodontal pocket has been central to mechanistic accounts linking periodontitis (PD) to cardiovascular disease (CVD) [8]. Within the SCZ spectrum, chronic inflammation and redox dysregulation are further associated with depleted AO defenses and pathological oxidative stress, which may aggravate core neurobiological abnormalities [9,10]; in parallel, oral pathogens may contribute upstream microglial/NOX-linked neuroinflammatory mechanisms [11]. In metabolic comorbidities such as diabetes mellitus (DM), this inflammatory–oxidative milieu can become bidirectionally reinforcing—raising susceptibility to periodontal disease (PD) and amplifying tissue vulnerability [12]—thereby deepening systemic “silent” inflammation. Because periodontal inflammation is accompanied by oxidative stress arising from an imbalance between reactive oxygen species (ROS) generation and AO defenses, periodontitis provides a clinically relevant context for applying foundational redox biology frameworks [13]. As established in core redox biology, the body’s three-line AO defense system—including superoxide dismutase (SOD), catalase (CAT), and glutathione peroxidase (GPx)—can become overwhelmed [14], shifting from eustress to oxidative distress and resulting in irreversible damage to DNA, lipids, and proteins [13,14].

Following the principle articulated by Steve Kisely [15]—“No Mental Health without Oral Health”—there is an urgent clinical need for integrated management that treats oral health as a core component of comprehensive care in severe mental illness (SMI) [15]. This objective is reinforced by the 2024 Mishu et al. consensus statement, which prioritizes reducing oral health inequalities in SMI and explicitly argues that oral health must be included in routine physical health assessment and care pathways [16]. In parallel, reverse-integration service delivery models—i.e., delivering collaborative physical healthcare within mental health settings—have been synthesized as pragmatic approaches to bridge structural gaps in care for adults with SMI [17]. Complementing these system-level models, implementation-focused strategies (such as link worker interventions to support routine dental visiting) are being tested to reduce access barriers and enable sustained engagement with dental services [18].

Historically, the systemic impact of PD has been framed through inflammatory spillover concepts, with periodontal–systemic associations consolidated in evidence syntheses addressing links between periodontitis and major non-communicable diseases [19] and pathogenic mechanisms connecting periodontitis with diabetes [20]. Building on these clinical foundations, mechanistic syntheses have clarified how oral dysbiosis can induce systemic immune responses and contribute to inflammatory comorbidities [21,22,23]. Consistent with this broader systemic perspective, umbrella-level evidence has linked periodontitis and tooth loss to higher risks of cognitive disorders [24]. Finally, recent physiology syntheses emphasize that persistent low-grade chronic inflammation constitutes a shared mechanistic substrate across major chronic diseases, with direct implications for prevention-oriented and risk-stratification frameworks [25].

The oral–systemic health connection has been a major focus of research, with roughly 47% of the work on this issue published between 2019 and 2024, consistent with a marked recent surge in publication output [26]. This shift is essential for preserving somatic longevity—by mitigating cardiovascular and metabolic risks—and may also be relevant for neurobiological function in PZ-spectrum conditions, where chronic peripheral inflammation is clinically associated with cognitive impairment [6]. Mechanistically, periodontitis-related microbial challenge can drive systemic inflammatory signaling [21], and oral pathogens can trigger microglial ROS and cytokine induction via NADPH oxidase 4 (NOX4)-dependent pathways [11]. Importantly, emerging periodontal-specific evidence indicates that mitochondrial deoxyribonucleic acid (mtDNA) efflux/release can occur in periodontal tissues and cells [27], providing a plausible route by which periodontal pathology may contribute pro-inflammatory danger signals beyond the oral niche. In broader chronic-disease contexts, mtDNA leakage has been discussed as a trigger of inflammation in age-related cardiovascular disease frameworks [28]. Together, these lines of evidence support reframing periodontitis not as a passive comorbidity, but as a biologically active distal source of systemic inflammatory and oxidative signaling with downstream implications for vascular and brain health [21,27,28].

Throughout this review, four population descriptors are used with deliberately distinct meanings and are not interchangeable. Psychosis (PZ) refers to the broad clinical phenotype encompassing positive, negative, and disorganization symptoms, irrespective of underlying diagnosis. Schizophrenia (SCZ) denotes the specific diagnostic entity defined by current DSM-5-TR or ICD-11 criteria. Schizophrenia-spectrum disorders (SSD) groups SCZ with schizoaffective disorder, schizophreniform disorder, and brief psychotic disorder, reflecting their shared neurobiological vulnerability. Severe mental illness (SMI) is a broader administrative and epidemiological category that additionally includes bipolar disorder with psychotic features and major depression with psychotic features. Whenever possible, we cite primary studies using the original terminology of the source publication; when integrating evidence across studies, the default scope of this review is SSD or PZ-spectrum populations. Periodontal-specific evidence (clinical attachment level, bleeding on probing, radiographic bone loss, and standard clinical staging and grading parameters [8,29]) is reported separately from broader oral health outcomes (tooth loss, caries, edentulism, access-to-care indicators) wherever the source data permit such disaggregation.

### 1.2. PD as a Marker of Oxidative Inflammatory Burden

PD is far more than a localized dental ailment; it is a complex, chronic inflammatory infection driven by a dysbiotic polymicrobial biofilm that provokes a destructive host immune response [8,21,29]. As a recognized source of systemic “inflammatory spillover,” PD has been linked to a broad range of non-communicable diseases, as summarized in landmark evidence reports addressing periodontal–systemic associations and diabetes-periodontitis links [19,20]. Building upon these clinical foundations, mechanistic syntheses have clarified how oral dysbiosis can elicit systemic immune responses [23,29], including through longer-range immunobiological “memory” frameworks that may be relevant to chronic comorbidity trajectories [30]. Evidence summarized in umbrella reviews suggests an association between periodontitis/tooth loss and a higher risk of cognitive disorders, although causal direction remains uncertain [24].

The biological mechanisms proposed to underpin these remote associations—including atherosclerotic cardiovascular disease (ACVD) [31,32] and DM [33,34]—include sustained translocation of microbial products and pro-inflammatory mediators into the circulation. Canonical periodontal pathogens such as *Porphyromonas gingivalis* (*P. gingivalis*) can release virulence factors (including gingipains), while microbial components such as lipopolysaccharides (LPS) can sustain systemic inflammatory and acute-phase responses characterized by elevated cytokines (e.g., interleukin-1β (IL-1β) and tumor necrosis factor-α (TNF-alpha) [8,21,23,29]. Consistent with this systemic risk framing, prospective evidence has further supported associations between periodontitis and cardiovascular mortality across population-based cohorts and clinically relevant subgroups [5,35,36].

Epidemiological data, including large-scale UK Biobank analyses, indicate that PD prevalence is elevated in individuals with a history of PZ compared to controls [37]. Findings from tertiary care settings likewise describe poorer periodontal indices and fewer remaining teeth (i.e., greater tooth loss burden) among psychotic patients relative to comparison groups [38]. Given its chronic and often insidious course, PD may contribute to sustained systemic oxidative inflammatory burden [21,39]. Furthermore, inflammatory cytokine responses after nonsurgical periodontal therapy (NSPT) can vary across periodontal stages and grades, underscoring heterogeneity in inflammatory outputs and potential systemic signaling [40]. In this context, periodontitis has been associated with lower total antioxidant capacity (TAC)—which can rise after NSPT—supporting TAC as an integrative marker of redox burden [41,42]. Complementing redox readouts, emerging geroscience-oriented work links periodontitis with biological age acceleration (e.g., biological age acceleration (BioAgeAccel)/phenotypic age acceleration (PhenoAgeAccel)) and suggests that accelerated biological aging may partially mediate associations between periodontal status and cognitive performance [36,43]. At the “omics” level, periodontitis shows reproducible metabolomic signatures across biofluids (saliva, gingival crevicular fluid (GCF), and plasma), including shifts involving amino acid and lipid-related metabolites; additionally, multi-omics studies suggest that periodontal therapy can induce partial re-alignment of microbial and metabolic outputs over time [44,45,46]. Finally, in psychiatric contexts, oral–brain axis literature and salivary microbiome–metabolome studies in SCZ support a plausible interface between periodontal status, dysbiosis, and disease-associated metabolic signaling—without implying direct causality [5,47].

### 1.3. Hypothesis and Rationale

We conducted a targeted literature search to support this critical narrative review. PubMed/MEDLINE and Google Scholar were searched for studies and reviews addressing (i) periodontitis/periodontal inflammation in SMI and PZ/SCZ, (ii) systemic inflammatory and redox biomarkers (e.g., C-reactive protein (CRP)/high-sensitivity C-reactive protein (hs-CRP), IL-6, 8-hydroxy-2’-deoxyguanosine (8-OHdG), F2-isoprostanes (F2-IsoPs), soluble receptor for advanced glycation end-products (sRAGE)/endogenously secreted RAGE (esRAGE), and (iii) mechanistic pathways linking metabolic stress to innate immune activation (e.g., AGEs–RAGE–nicotinamide adenine dinucleotide phosphate oxidase (NOX), thioredoxin-interacting protein (TXNIP)–NLR family pyrin domain-containing 3 (NLRP3), mitochondrial damage-associated molecular pattern (DAMPs) signaling. Priority was given to high-level evidence (systematic reviews/meta-analyses), interventional periodontal therapy studies reporting systemic outcomes, and recent mechanistic reviews in immunology and redox biology. Because this manuscript is a critical narrative synthesis rather than a systematic review, Preferred Reporting Items for Systematic Reviews and Meta-Analyses (PRISMA)-based screening and flow reporting were not performed; nevertheless, the synthesis consistently differentiates observational associations from interventional evidence and mechanistic hypotheses.

#### 1.3.1. Distinguishing Hypothesis from Evidence

Throughout this critical narrative synthesis, we explicitly separate (1) observational associations (cross-sectional/cohort evidence linking PD to systemic inflammatory/redox markers and to PZ-relevant outcomes), (2) interventional evidence (changes in systemic biomarkers following NSPT), and (3) mechanistic plausibility (redox–immune pathways such as AGE–RAGE–NOX activation, TXNIP–NLRP3 inflammasome signaling, and mitochondrial DAMPs-driven innate immune activation). Causal language is restricted to evidence supported by randomized trials and meta-analyses of interventions, while mechanistic sections are framed as testable pathways and hypotheses. This review synthesizes extensive epidemiological data with the molecular underpinnings of systemic and psychiatric disorders. We propose that the association between PD and PZ is sustained by a multifactorial “pathological confluence” rather than solely by shared genetic liability. In line with this framing, two-sample Mendelian randomization did not provide evidence for a bidirectional genetic causal relationship between genetically predicted periodontitis and common psychiatric disorders [48], suggesting that non-genetic and potentially modifiable factors (e.g., acquired inflammatory exposures, behaviors, and healthcare mediators) may contribute to observed epidemiological links [48]. Backward citation tracking (screening the reference lists of key papers) was used to identify additional sources [49].

In this model, PD acts as a significant acquired inflammatory trigger that can exacerbate systemic inflammatory and oxidative–metabolic vulnerability relevant to PZ, consistent with evidence linking PZ with poorer oral health and with evidence connecting chronic peripheral inflammation to cognitive impairment in SCZ-spectrum disorders [6,50]. This intensification involves the production of ROS and reactive nitrogen species (RNS), which, when insufficiently counterbalanced by the body’s three-line AO defense system, can lead to oxidative distress and biomolecular damage [13,14,51].

Consequently, professional periodontal treatment—specifically Scaling and Root Planing (SRP)/NSPT—should be considered a core component of integrated somatic care pathways for people with SMI, given the systemic inflammatory spillover of periodontitis and the disproportionate burden of preventable comorbidity. This position aligns with the global consensus that PD is independently associated with systemic non-communicable diseases and warrants a shift from a purely localized approach to a systemic management framework [52]. Moreover, evidence indicates that periodontal therapy can reduce systemic inflammatory burden, including CRP [53,54], and can improve glycaemic control as reflected by reductions in glycated hemoglobin (HbA1c) in people with diabetes [55]. Additionally, a recent meta-analysis implies that NSPT might be linked to modest reductions in HbA1c levels even among non-diabetic periodontitis patients, suggesting that the resolution of periodontal inflammation exerts systemic metabolic effects extending beyond diabetic cohorts [36]. However, because the available evidence in non-diabetic cohorts is largely non-randomized and self-controlled, these estimates should be interpreted as hypothesis-supporting signals rather than definitive causal effects. Quantitatively, evidence synthesis suggests that NSPT reduces systemic CRP at ~6 months, while multicenter evidence in systemically healthy individuals indicates decreases in IL-6/hsCRP following periodontal treatment, and meta-analytic data suggest modest HbA1c reductions even in non-diabetic periodontitis—albeit based largely on non-randomized/self-controlled evidence (see Supplementary Table S1).

#### 1.3.2. Interpretive Guardrails

For systemic outcomes, we report the direction and magnitude of effects where meta-analytic estimates are available and explicitly acknowledge heterogeneity, baseline-risk dependence, and limitations of study design (e.g., self-controlled case series). Claims regarding PZ relevance are framed as plausibility and risk-modifying contexts rather than direct causal inference. For transparency, key systemic biomarker effects of NSPT cited in this section are summarized in Supplementary Table S1.

In parallel, efforts to reduce oral health inequalities in SMI support integrated care models and pragmatic service interventions that facilitate access to dental services [16,18]. By attenuating this distal source of inflammatory spillover—the proposed chronic source of systemic inflammatory spillover—periodontal interventions may help reduce the inflammatory–metabolic vulnerability that contributes to preventable comorbidity and premature mortality in PZ-spectrum populations [2,4]. This clinical framing is further supported by evidence that periodontal treatment can reduce chronic inflammation even in systemically healthy periodontitis patients [56].

#### 1.3.3. Search Traceability

The targeted search was last updated on 8 March 2026. We queried two complementary sources: PubMed/MEDLINE (primary database, used for systematic retrieval and structured screening) and Google Scholar (auxiliary source, used only for citation tracking and to identify additional primary studies cited within retrieved reviews). For PubMed, we combined MeSH headings and free-text keywords across two query blocks: Block A (periodontal exposure × psychosis/schizophrenia/severe mental illness) and Block B (periodontal exposure × oxidative stress/NLRP3 inflammasome/AGEs–RAGE/mitochondrial/NOX4). Filters included English language, 2000 to 8 March 2026, humans where applicable. The complete query strings for both blocks, together with the screening workflow, inclusion/exclusion rules, and citation tracking procedure, are provided in Supplementary Table S2 and are intended to be fully reproducible by an independent reader.

Google Scholar was not used for primary database screening because of its non-deterministic ranking and lack of structured filters. Its role was strictly limited to the following: (a) backward citation tracking of key reviews and primary studies retrieved through PubMed; (b) forward citation tracking of seminal mechanistic papers (TXNIP–NLRP3, AGEs–RAGE, NOX4-microglia, mtDNA-DAMP); and (c) targeted retrieval of full texts when PubMed indexing was incomplete. Records identified through Google Scholar were subsequently verified in PubMed/Crossref and processed through the same inclusion/exclusion criteria applied to the primary search.

Study prioritization followed an explicit hierarchy aligned with the evidence levels used in Table 1. For treatment effect statements (e.g., NSPT effects on CRP, IL-6, HbA1c), we prioritized Cochrane reviews, systematic reviews with meta-analysis, and randomized controlled trials, supplemented by large prospective cohorts when interventional evidence was unavailable. For epidemiological associations (e.g., periodontitis prevalence in PZ/SMI), we prioritized population-level cohort studies, registry data, and Mendelian randomization analyses over cross-sectional surveys. For mechanistic claims (redox/inflammatory pathways), we prioritized primary experimental studies in human-relevant models (human gingival fibroblasts, human neural progenitors, ex vivo periodontal tissue, microbiome-defined preclinical models) over secondary narrative reviews; reviews were cited only when they consolidated convergent primary evidence or provided a pathway-level context that no single primary study covered. When a single high-quality primary study and a recent systematic review supported the same claim, both were cited; when only narrative reviews were available, the claim was downgraded to the ‘Mechanistic plausibility’ or ‘Integrative hypothesis’ tier (see Table 1).

**Table 1:** Evidence-level synthesis of the principal claims advanced in this review. Each claim domain is mapped to one of four evidence tiers (observational, interventional/RCT, mechanistic preclinical, integrative hypothesis) together with the strength of human evidence available in PZ-spectrum populations. The table is intended to make explicit the calibration between mechanistic plausibility and clinically demonstrated causality, in line with the cautious interpretive frame adopted throughout this manuscript.

Claim Domain	Evidence Tier	Key References	Strength of Human Evidence in PZ-Spectrum
PD prevalence and severity in PZ-spectrum populations	Observational (cross-sectional, case–control, cohort)	[37,50,57]	Established association; effect-size heterogeneity; residual confounding plausible
NSPT effects on systemic CRP, IL-6, and HbA1c	Interventional (RCT meta-analyses)	[53,55] (Supplementary Table S1)	Established short- to medium-term clinical effect outside PZ; not yet replicated within PZ-spectrum cohorts
Mendelian randomization of periodontitis × major psychiatric disorders	Genetic epidemiological	[48]	Negative for bidirectional genetic causality; supports acquired pathway interpretation
TXNIP–NLRP3 inflammasome axis as a metabolic redox node	Mechanistic (preclinical + extrapolated human)	[58,59]	Mechanistically plausible; direct human confirmation in PZ-spectrum populations pending
AGEs–RAGE axis sustaining a self-perpetuating ROS loop	Mechanistic (preclinical + non-psychiatric clinical)	[60,61,62]	Mechanistically plausible; PZ-specific human data limited
NOX4-linked microglial activation by *P. gingivalis*	Preclinical (in vivo + in vitro)	[11,23]	Preclinically supported; no human PZ-specific replication
mtDNA leakage and DAMP-mediated sterile inflammation	Mechanistic (preclinical, cross-tissue)	[28]	Mechanistically plausible; not validated in PZ tissues
BBB permeability via Mfsd2a/Caveolin-1 axis under *P. gingivalis* bacteremia	Preclinical (animal model)	[63,64]	Preclinically supported; human imaging/CSF correlates pending
NAC as an adjunctive redox modulator in SCZ	Interventional (RCTs + meta-analysis)	[65,66]	Signal of benefit in negative symptoms and cognition; replication needed; not periodontitis specific
MitoQ in early SCZ-spectrum populations	Translational/early-phase clinical	[67,68]	Feasibility supported by preclinical rationale and trial listings; clinical efficacy not yet established
Salivary NLRP3 and GCF cytokine profiles after NSPT	Observational (clinical biomarker)	[69,70]	Emerging clinical signal in periodontitis; not yet integrated with PZ phenotyping
Periodontitis as a plausible upstream amplifier of systemic redox burden in PZ	Integrative hypothesis (this review)	Synthesis	Hypothesis generating, requires prospective and interventional human evidence
Oral–systemic–brain axis converging on TXNIP–NLRP3	Integrative hypothesis (this review)	Synthesis (Figure 1)	Hypothesis generating, supported by convergent preclinical and observational lines

Abbreviations: AGEs, advanced glycation end-products; BBB, blood–brain barrier; Caveolin-1, caveolae-associated protein 1; CRP, C-reactive protein; CSF, cerebrospinal fluid; GCF, gingival crevicular fluid; HbA1c, glycated hemoglobin; IL-6, interleukin-6; Mfsd2a, major facilitator superfamily domain-containing protein 2A; MitoQ, mitoquinone mesylate (mitochondria-targeted antioxidant); mtDNA, mitochondrial DNA; NAC, N-acetylcysteine; NLR, NOD-like receptor; NLRP3, NLR family pyrin domain-containing protein 3; NOX4, NADPH oxidase 4; NSPT, nonsurgical periodontal therapy; PD, periodontitis (also referred to as periodontal disease); PZ, psychosis; RAGE, receptor for advanced glycation end-products; RCT, randomized controlled trial; SCZ, schizophrenia; TXNIP, thioredoxin-interacting protein.

**Figure 1:**
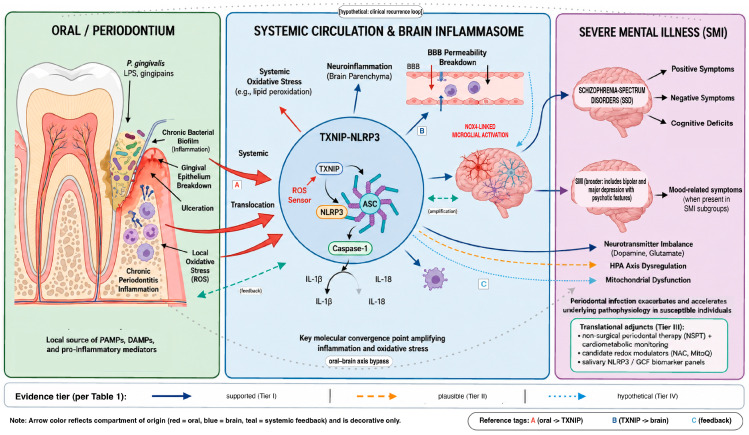
Oral–systemic–brain pathway linking periodontitis (PD) and psychosis (PZ) via immunometabolic redox stress and the TXNIP–NLRP3 node. The schematic depicts three interacting compartments. ((**Left**), green—“Oral/periodontium”): Chronic bacterial biofilm dominated by *Porphyromonas gingivalis* (releasing lipopolysaccharide and gingipain virulence factors), gingival epithelium breakdown, ulceration, and chronic periodontitis-associated local oxidative stress (reactive oxygen species, ROS), constituting a continuous local source of pathogen- and damage-associated molecular patterns (PAMPs/DAMPs) and pro-inflammatory mediators. ((**Center**), light blue—“Systemic circulation and brain inflammasome”): Oral signals and systemic redox stress converge on the TXNIP–NLRP3 inflammasome (TXNIP–ROS sensor/NLRP3–ASC (apoptosis-associated speck-like protein containing a CARD)/caspase-1 axis), driving maturation of interleukin (IL)-1beta and IL-18, sustaining systemic oxidative stress (e.g., lipid peroxidation), promoting blood–brain barrier (BBB) permeability and breakdown, and supporting NOX4-linked microglial activation and neuroinflammation in the brain parenchyma; this compartment represents the key molecular convergence point amplifying inflammation and oxidative stress. ((**Right**), lavender—“Severe mental illness (SMI)”): Schizophrenia-spectrum disorders (SSD)—including positive symptoms, negative symptoms, and cognitive deficits—together with mood-related symptoms when present in broader SMI subgroups (bipolar disorder and major depression with psychotic features), neurotransmitter imbalance (dopamine, glutamate), hypothalamic–pituitary–adrenal (HPA) axis dysregulation, and mitochondrial dysfunction. A dedicated bottom-right inset summarizes translational adjuncts (Tier III)—nonsurgical periodontal therapy (NSPT) combined with cardiometabolic monitoring, candidate redox modulators (N-acetylcysteine, NAC; mitoquinone mesylate, MitoQ), and oral fluid biomarker panels (salivary NLRP3, gingival crevicular fluid cytokines)—positioned as the actionable layer of the cascade. Arrow styles encode evidence tier (per Table 1): solid arrows = supported (Tier I—RCTs, meta-analyses, large cohorts), with reference labels [8,21,23] for the supported oral-to-systemic axis and [59] for the TXNIP–NLRP3-to-brain axis; dashed arrows = plausible (Tier II—convergent mechanistic evidence), including the bidirectional feedback loop between the oral and systemic compartments (“feedback”) and the bidirectional amplification loop between the systemic and brain compartments [59,69] (“amplification”); dotted arrows = hypothetical (Tier IV—extensions proposed by the present review), including the direct oral–brain axis bypass [11,28] in the lower margin and the curved clinical recurrence feedback loop in the upper margin. Arrow color reflects compartment of origin (red = oral, blue = brain, and teal = systemic feedback) and is decorative only; the level of evidence is encoded exclusively by arrow style (solid/dashed/dotted), as detailed in the legend bar at the bottom of the figure. Reference tags A–C in the figure summarize the three principal supported/plausible links (A: oral TXNIP–NLRP3 [8,21,23]; B: brain TXNIP–NLRP3 [59]; C: bidirectional feedback [59,69]). Abbreviations: BBB, blood–brain barrier; CNS, central nervous system; CRP, C-reactive protein; DAMPs, damage-associated molecular patterns; GCF, gingival crevicular fluid; HPA, hypothalamic–pituitary–adrenal; IL, interleukin; MitoQ, mitoquinone mesylate; NAC, N-acetylcysteine; NF-kB, nuclear factor κ-B; NLRP3, NOD-like receptor pyrin domain-containing 3; NOX4, NADPH oxidase 4; NSPT, nonsurgical periodontal therapy; PAMPs, pathogen-associated molecular patterns; PD, periodontal disease; PZ, psychosis; ROS, reactive oxygen species; SMI, severe mental illness; SSD, schizophrenia-spectrum disorders; TXNIP, thioredoxin-interacting protein. Conceptual schematic prepared with Google Gemini AI assistance for the graphical layout and verified by all co-authors.

Each principal claim of this review was explicitly mapped to one of four evidence tiers and is reported in Table 1 (Evidence-level synthesis): Tier I (observational/interventional human evidence)—claims supported by RCTs, systematic reviews, or large cohorts in human PZ-spectrum or periodontitis populations; Tier II (mechanistic plausibility)—claims supported by convergent in vitro and animal evidence, with no direct human interventional confirmation; Tier III (translational adjuncts)—candidate therapeutic interpretations supported by preclinical pharmacology and limited early-phase human data; Tier IV (integrative hypothesis)—synthesis-level propositions formulated by the present review, explicitly framed as hypothesis generating. The verbal hedging used throughout the manuscript (‘may’, ‘plausibly’, ‘candidate’, ‘hypothesis-generating’) is calibrated to the corresponding tier so that a reader can trace any in-text claim to its evidence anchor in Table 1 and, where relevant, to the systemic biomarker effects summarized in Supplementary Table S1.

Records were excluded when they were off-topic (no periodontal exposure or no PZ/SMI relevance), did not report outcomes relevant to the specific claim being made, were not available in full text when critical details were required, or represented duplicate/overlapping publications without incremental contribution. This work is a critical narrative review rather than a formal systematic review, and therefore did not implement a full PRISMA workflow; it does, however, follow SANRA-style transparency principles. Supplementary Tables S1 and S2 are essential to reproducibility and interpretability and are closely aligned with the in-text statements: Table S1 lists every primary study cited in support of NSPT systemic biomarker claims (with design, population, effect size, 95% CI, and limitations), while Table S2 documents the full search strategy, screening and selection guardrails, and citation tracking procedure.

## 2. Epidemiological Convergence and the Pursuit of Etiology

### 2.1. Consistency of Observational Data and Associated Risk Factors

The increasing prevalence and burden of PD in individuals with PZ—as reported in large population-based analyses [37] and corroborated by case–control evidence linking SCZ-spectrum disorders to poorer oral health outcomes [50]—underscores a pattern of overlapping vulnerability with chronic systemic disease. Collectively, these epidemiological findings support the interpretation of the periodontium as a clinically accessible correlate of broader systemic perturbations in psychiatric populations, including low-grade inflammation and immune dysregulation [2]. Convergent metabolomic evidence further strengthens this framework by documenting metabolomic alterations relevant to psychiatric disorders and SCZ, consistent with the view that systemic metabolic reprogramming is a pervasive feature across the PZ-spectrum [5,71].

### 2.2. Behavioral and Substance Use Drivers

Smoking remains a central modifiable exposure shaping PD risk and clinical burden, and it is especially relevant in people with psychotic disorders treated with antipsychotics, where smoking frequently co-occurs with medication-related oral adverse effects and compromised oral self-care. In a cross-sectional study of patients with SCZ taking antipsychotic medication, severe PD was explicitly linked to medication side effects, poor dental hygiene, and smoking [72].

At the population level, UK Biobank data indicate that PD is more prevalent among individuals with a history of PZ and that smoking is among the lifestyle factors associated with PD in this group [37]. These findings support integrating tobacco cessation components into oral health prevention and early-detection programs tailored to serious mental illness.

From a broader periodontal–systemic perspective, evidence syntheses highlight substantial heterogeneity in periodontitis definitions and generally report modest associations between periodontitis and several chronic conditions, emphasizing careful interpretation of systemic links while still accounting for shared behavioral and metabolic risk pathways [19].

Beyond behavioral clustering, tobacco smoke represents an intense toxic exposure: CDC materials state that commercial tobacco smoke contains more than 7000 chemicals, including about 70 that can cause cancer [73]. This evidence supports framing smoking as a plausible pro-oxidative and pro-inflammatory driver that can amplify periodontal tissue injury, particularly in already vulnerable clinical populations.

Other substance use exposures may further compound vulnerability. For khat (Catha edulis), a meta-analysis discussing student use notes reported associations in the literature with adverse outcomes, including PZ, cardiovascular problems, poor oral hygiene, and PD [74]. While this source is primarily epidemiological, it provides a defensible rationale for considering khat exposure in risk profiling when clinically relevant.

Regarding alcohol, systematic evidence in alcohol use disorder indicates that chronic exposure is associated with oxidative stress and impairment of AO defenses [75]. Mechanistic evidence also supports a mitochondria-centered pathway: chronic alcohol exposure alters mitochondrial calcium handling and can increase mitochondrial ROS generation in hepatocytes under Ca^2+^-mobilizing stimulation [76]. Together, these findings provide a coherent biological basis for discussing chronic alcohol use as a contributor to redox imbalance and inflammation, which may plausibly aggravate periodontal vulnerability in susceptible patients.

In the broader cardiometabolic context, metabolic syndrome is described as a cluster of abnormalities linked with oxidative stress, inflammation, and metabolic dysregulation, with discussion of mitochondrial ROS and AO defense systems [77]. Such a framework can be used to justify why chronic redox stressors (including sustained alcohol exposure) may interact with systemic metabolic risk to worsen periodontal inflammatory trajectories.

Finally, emerging work on mitochondrial danger signaling offers a mechanistic model for sustained systemic inflammation: increased mitochondrial DNA (mtDNA) leakage in senescent contexts may activate innate immune pathways, including cyclic GMP–AMP synthase–stimulator of interferon genes (cGAS–STING), toll-like receptor 9 (TLR9), and inflammasomes [28]. This “mitochondrial DAMPs” framework supports the plausibility of oxidative/mitochondrial stress sustaining chronic inflammation relevant to cardiometabolic disease and potentially intersecting with periodontal inflammatory burden.

### 2.3. Systemic Comorbidities as Inflammatory Indicators

Comorbid cardiometabolic disease may amplify periodontal vulnerability in people with PZ through shared, acquired risk pathways (e.g., smoking, metabolic dysregulation, and multimorbidity) rather than a primary genetic causal link. In a large UK Biobank analysis, PD was more prevalent in individuals with a history of PZ than in the general population, and, within the PZ subgroup, PD was associated with smoking and with comorbid cardiovascular, cancer, or respiratory disease, supporting the clinical relevance of multimorbidity when interpreting periodontal burden in PZ [37].

In NHANES-based analyses, severe mental illness was linked to poorer oral health outcomes after multivariable adjustment, including tooth loss (OR 1.60, 95% CI 1.34–1.92), mouthache (OR 1.42, 95% CI 1.11–1.82), and dental caries counts (RR 1.14, 95% CI 1.05–1.23) [1]. Diabetes and smoking history were key predictors of tooth loss within SMI (e.g., diabetes OR 1.86, 95% CI 1.07–3.26), while PD severity did not differ significantly between SMI and non-SMI in the available periodontal subsample [1].

In parallel, Mendelian randomization (MR) data do not support a primary bidirectional genetic causal relationship between periodontitis and common psychiatric disorders, implying that at least part of the epidemiological overlap between psychiatric illness and PD is more consistent with confounding and downstream exposures (e.g., behaviors, medication effects, and comorbid medical disease) than with direct genetic causality [48].

At the molecular level, convergent metabolomics literature in psychiatric disorders reports systemic metabolic alterations spanning lipid classes (including lysophosphatidylcholines) and dysregulated amino acid pathways relevant to neurotransmitter metabolism, consistent with a broader immune–metabolic imbalance that can plausibly interact with cardiometabolic comorbidity and inflammatory burden [71].

Clinical case–control evidence supports that oral inflammatory burden and oral hygiene impairment are common in psychiatric cohorts and track with modifiable and treatment-related factors. Psychiatric groups showed higher plaque burden and gingival inflammation (e.g., plaque index median 2 vs. 1 in controls; BOP ~17–20% vs. 3% in controls), with markedly higher smoking prevalence (~45% vs. 9.6% in controls) and an additional signal for treatment complexity (polypharmacy) worsening plaque and bleeding indices; however, evidence for clinically meaningful differences in probing-depth outcomes was limited in multivariable models [50].

This oral–systemic vulnerability is plausibly reinforced by the chronic low-grade inflammatory and immune–metabolic dysregulation described across psychiatric disorders and metabolic syndrome frameworks [2], and by guideline-recognized cardiometabolic risk pathways during antipsychotic treatment—including smoking, alcohol misuse, excessive weight gain, and diabetes—together with the practical challenge that monitoring and preventive interventions are frequently inadequate in real-world care [4].

Mechanistically, cardiometabolic comorbidity can sustain inflammatory tone through mitochondrial danger signaling. mtDNA leakage can function as an inflammatory trigger engaging innate immune pathways (including cGAS–STING, TLR9, and inflammasome activation), providing a biologically plausible bridge between systemic inflammatory burden and downstream tissue injury relevant to oral–systemic interactions [28].

Consistent with this framework, trajectory-based evidence identifies a high-risk LBRI trajectory during early second-generation antipsychotics (SGAs) treatment in first-episode SCZ (e.g., ~6% of patients; ~+3.5 kg/m^2^ within the first 3 months), which supports early cardiometabolic risk stratification—particularly in olanzapine-treated individuals [5]. Crucially, longitudinal first-episode PZ data measuring inflammation show that hs-CRP increases over the first treatment year alongside marked anthropometric changes, with waist circumference emerging as a significant predictor of hs-CRP—supporting the interpretation that early abdominal adiposity development is accompanied by chronic low-grade inflammation in this population [78].

### 2.4. Socioeconomic and Access Barriers

Low socioeconomic status (SES)—often reflected in deprivation indices and lower educational attainment—can act as a persistent confounder in the PZ–PD relationship because it shapes both upstream cardiometabolic risk and downstream access to preventive dental care. In UK Biobank analyses of PZ history and PD, socioeconomic position is embedded in the covariate structure (e.g., deprivation measures) and is therefore critical for interpreting observed associations [37]. Educational attainment also appears clinically relevant for metabolic vulnerability: in first-episode SCZ, lower education was independently associated with membership in the high-risk LBRI trajectory (odds ratio (OR) 5.40), which may amplify susceptibility to cardiometabolic dysregulation that can co-occur with poorer oral health outcomes [5]. In parallel, early treatment-year adiposity is accompanied by measurable low-grade systemic inflammation (hs-CRP), which rises during follow-up and is predicted by waist circumference, supporting a biologically plausible “social → adiposity → inflammation” amplification route [78]. More broadly, SES-linked constraints can limit access to nutritious diets, regular physical activity, and timely oral healthcare, thereby amplifying established oral health inequalities in SMI populations [15]. Consistent with this framing, large population analyses in SMI show that a lower income is associated with worse oral outcomes (including tooth loss and poorer self-rated oral health), supporting the inclusion of SES/access factors as central—not peripheral—determinants in oral–mental health comorbidity models [1].

Patients with SMI commonly face layered barriers to dental care spanning patient-, provider-, and system-level determinants, including practical constraints (appointment planning, transport, costs), discontinuity of care, and relational/communication factors in dental settings [79]. Psychological and practical barriers—such as dental anxiety and competing priorities—can further reduce routine dental visiting and adherence to sustained oral hygiene recommendations, while clinical complexity (e.g., smoking and polypharmacy) may exacerbate downstream oral risk [50]. Accordingly, cross-sector collaboration between mental health and dental services has been proposed and operationalized via structured “link worker” approaches designed to provide practical navigation support (booking/planning/attendance assistance), anxiety management, and motivational reinforcement to increase routine dental visiting [18]. Complementing service–navigation interventions, a rights-based, consensus-driven action plan emphasizes that oral health should be embedded in physical health assessment pathways in SMI, that access to dental services must improve, and that oral health should be reflected in healthcare training and systems [16]. Feasibility trial evidence further indicates that link work models can be delivered with high engagement and show signals of improved dental attendance when supported by dental record linkage approaches [80].

Finally, Mendelian randomization analyses do not support a bidirectional genetic causal relationship between periodontitis and major psychiatric disorders, strengthening the interpretation that elevated PD burden in PZ-spectrum populations is more plausibly driven by acquired pathways—social disadvantage, health behaviors, comorbidity clustering, and care access barriers—rather than shared genomic predisposition [48].

Instead, the association is better framed as an acquired multifactorial framework shaped by environmental stressors, behavioral determinants, and treatment-related iatrogenesis rather than as a primarily shared genetic predisposition. This shift redirects mechanistic and clinical research away from genetic screening alone and toward modifiable, acquired biological processes—including cardiometabolic dysregulation and systemic inflammatory/oxidative load—that may accumulate in psychiatric populations [2,10].

Longer-term follow-up studies in early SCZ also document concurrent worsening of metabolic status and inflammatory biomarker profiles (including IL-6 among other cytokines) during chronic illness/treatment trajectories, consistent with progressive immunometabolic strain [57]. From a systems biology perspective, this increases interest in metabolomic biomarker clusters (including lipid and amino acid disturbances) reported across psychiatric disorders, which may reflect broader systemic dysregulation relevant to somatic comorbidity and risk clustering [71].

Given that genomic evidence for causality remains unsupported by Mendelian randomization, greater attention should be paid to the adverse effects of antipsychotic treatment and other acquired pathways that can plausibly worsen oral health trajectories. Psychotropic medications can contribute to xerostomia (reduced salivary flow), weakening an important mechanical and biochemical barrier and increasing susceptibility to oral disease processes [1]. Clinical case–control evidence also documents poorer oral parameters (notably plaque accumulation and gingival inflammation/bleeding indices, alongside caries experience) in PZ-spectrum and severe affective disorder groups, with behavioral factors such as smoking further aggravating risk [50].

Beyond local oral disruption, systemic metabolic dysregulation is a major contributor to somatic risk in PZ and antipsychotic-treated populations. Clinical guidance and reviews highlight that antipsychotic exposure is associated with weight gain and metabolic disturbances that elevate cardiometabolic risk [4,81]. In populations with type 2DM, meta-analyses indicate that periodontal therapy is associated with reductions [82] in systemic inflammatory markers, including CRP and TNF-α [82]. Oxidative stress is also a relevant biological domain both for SCZ-spectrum pathophysiology and for PD activity, supporting the plausibility that combined metabolic and oxidative burden may accelerate tissue vulnerability [10,13]. Consistent with an oral–systemic inflammatory axis, periodontitis is associated with higher circulating CRP/hs-CRP, and periodontal therapy can reduce hs-CRP over follow-up [83].

Finally, reconceptualizing PD burden in SMI as modifiable and acquired (rather than genetically fixed) aligns with practical avenues for reversibility via service redesign and supported access. In this context, oral health integration and targeted support (including link worker approaches) are repeatedly emphasized as actionable strategies to reduce inequalities and improve dental visiting in SMI populations [15,18,80].

### 2.5. Confounding and Interpretative Caveats

The associations between PD and PZ-spectrum disorders described above are predominantly observational and are subject to substantial residual confounding. Five clusters of potential confounders deserve explicit consideration. First, lifestyle factors—particularly tobacco smoking, alcohol use, and substance use—are markedly more prevalent in PZ-spectrum populations and are themselves established drivers of periodontal destruction, oxidative burden, and systemic low-grade inflammation [3,16,36]. Second, structural determinants (socioeconomic status, educational attainment, deprivation indices, and access to dental and general medical care) shape both periodontal phenotype and psychiatric trajectory through shared upstream pathways and may attenuate any putative direct biological link [16,57,79].

Third, antipsychotic exposure—including drug class (first- vs. second-generation classes), specific agent, dose, and treatment duration—exerts heterogeneous effects on salivary flow, metabolic status, and prolactin signaling, and has been repeatedly implicated in the iatrogenic component of oral and systemic morbidity in PZ [4,84,85]. Fourth, cardiometabolic comorbidities (metabolic syndrome, type 2 diabetes mellitus, obesity, dyslipidemia) cluster with PZ and with periodontitis, raising the possibility that part of the observed association is explained by shared metabolic confounding rather than by an independent oral–brain biological axis [2,85,86,87]. Fifth, multimorbidity, polypharmacy, and fragmented oral hygiene access are independent contributors to oral health deterioration in this population and should be considered when interpreting cross-sectional associations [82].

Two complementary lines of evidence help to constrain interpretation. Mendelian randomization analyses do not currently support a bidirectional genetic causal relationship between periodontitis and major psychiatric disorders [48], reinforcing the view that the observed association reflects acquired rather than genetically fixed pathways. At the same time, the biological plausibility of redox- and inflammasome-mediated mechanisms (Section 3.2.4 and Section 3.3) means that confounding cannot fully explain the totality of the convergent observational, preclinical, and biomarker-based findings. We therefore frame the periodontitis–PZ relationship as a partially confounded but biologically grounded association, in which residual confounding by smoking, alcohol and substance use, socioeconomic status, antipsychotic exposure, metabolic syndrome, oral hygiene access, and multimorbidity may attenuate, mediate, or partially explain the observed link, while not exhausting it.

## 3. Mechanisms of Iatrogenic, Metabolic, and Inflammatory Convergence

### 3.1. Antipsychotic Iatrogenesis: The Dual Threat

#### 3.1.1. Salivary Dysfunction and Redox Imbalance

A convergent multifactorial framework can be used to conceptualize the PD–PZ connection as a convergence of systemic inflammatory signaling and oxidative stress pathways. Periodontitis is increasingly viewed as a dysbiotic inflammatory condition with the potential to influence extra-oral physiology through immune subversion and sustained inflammatory tone, providing a biologically plausible route for bidirectional amplification with neuropsychiatric vulnerability. Within SCZ-spectrum disorders, oxidative stress is repeatedly implicated in pathophysiology and symptom expression, and low-grade systemic inflammation (e.g., CRP elevation) provides an additional shared axis that can integrate oral- and brain-centered models [10,21,88].

Antipsychotic treatment can contribute iatrogenically to oral risk by disrupting salivary homeostasis (subjective xerostomia and/or objective hyposalivation), thereby weakening oral clearance, buffering capacity, and mucosal defense—changes that may increase vulnerability to gingival inflammation and periodontal deterioration. Epidemiologic data in SMI show a disproportionate burden of adverse oral outcomes (including tooth loss and periodontitis-related measures), supporting the clinical relevance of oral risk profiling in this population [1]. In particular, in a population-based retrospective cohort of newly diagnosed SCZ, antipsychotic exposure was associated with a high incidence of treated PD within one year, and hyposalivation (reported as an adverse effect linked to medication exposure) was associated with increased periodontal risk [1,89].

At the same time, salivary dysfunction in PZ is not unidirectional: clozapine frequently induces hypersalivation/sialorrhea, a paradoxical and clinically significant adverse effect that can impair sleep, social functioning, and adherence, and therefore warrants active monitoring and management. Mechanistic and therapeutic considerations for clozapine-induced sialorrhea have been synthesized in earlier reviews [84,90], and randomized trial evidence has been consolidated in a systematic review and meta-analysis focused on treatment strategies for clozapine-induced sialorrhea [84,90,91].

Given these medication- and disease-related pressures on oral physiology, salivary pH and unstimulated salivary flow rate represent practical, measurable parameters that can be followed longitudinally alongside periodontal indices to support individualized oral risk monitoring. Evidence from a clinical cohort indicates that lower salivary pH and reduced flow rate track with PD severity and can change measurably after oral hygienization, supporting feasibility as adjunct readouts in periodontal risk assessment [92].

Finally, PD is consistently associated with redox dysregulation—manifesting as increased oxidative damage markers and altered AO capacity in saliva and blood—supporting oxidative stress amplification within periodontal tissues and potentially beyond. For example, chronic periodontitis has been associated with significantly elevated salivary (and serum) lipid peroxidation (malondialdehyde (MDA)) with modest reductions in TAC [13]. Within a mechanistic framing of AO defense, disruption or insufficiency of first-line enzymatic systems—especially SOD, CAT, and GPx—shifts biology toward a pro-oxidant milieu [14], while contemporary redox biology language emphasizes disturbed redox regulation/homeostasis as a cross-disease organizing principle [51]. These redox mechanisms align with SCZ-spectrum models in which oxidative stress is positioned as a contributor to illness progression and outcomes [10,13,14,51].

#### 3.1.2. The Oxidative Burst and Systemic AO Exhaustion

Subgingival dysbiotic biofilms in periodontitis sustain intense polymorphonuclear neutrophil (PMN) recruitment. Viana et al. (2025) describe how neutrophils contribute to periodontal pathology through core effector programs that include ROS generation via the respiratory/oxidative burst, among other antimicrobial and inflammatory mechanisms [93]. In addition, aging-related changes in neutrophil chemotaxis, activation, antimicrobial function, and lifespan may modulate susceptibility and overall disease impact in periodontitis [5]. At a mechanistic level, ROS-driven periodontal tissue injury can be understood as a “double-edged” process—necessary for antimicrobial defense but potentially destructive when excessive or persistent—through pathways such as NF-κB/MAPK and inflammasome-linked mechanisms [94]. In the clinical measurement context, Mousa et al. (2024) emphasize the relevance of neutrophil-derived ROS to periodontal tissue destruction and report that ROS levels correlate positively with clinical attachment loss (CAL), supporting a disease-severity association [95]. Framed in modern redox biology, pathology emerges when redox regulation fails, and oxidant pressure exceeds AO control, shifting the system toward oxidative distress [51]; consistent with this positioning, oxidative stress is also discussed as a central pathogenic amplifier and a difficult (often therapeutically unsatisfactory) target in periodontitis-focused overviews [96].

Subgingival dysbiosis not only recruits but can also prime neutrophils; consequently, their oxidative (respiratory) burst becomes a major local source of ROS (superoxide (O_2_^•−^) and hydrogen peroxide (H_2_O_2_)), and when persistent, this antimicrobial response can intensify collateral host tissue injury [93,94]. Notably, Aboodi et al. (2016) support the broader premise that gingival inflammation is accompanied by increased oral neutrophil recruitment and coordinated shifts in salivary inflammatory/resolution signatures—a useful context for “neutrophil influx” framing—although this pilot study is not a primary source for specifying ROS or oxidative burst biochemistry [97]. When ROS generation exceeds AO buffering capacity, this imbalance aligns with the oxidative distress framing in redox biology [51]. Consistent with this redox shift, interventional human studies report reduced systemic AO defenses in PD, including lower serum TAC/related AO measures versus healthy controls, with improvement in at least some systemic AO indices after nonsurgical periodontal therapy in periodontitis [98]. In parallel, salivary TAC can increase after periodontal treatment in chronic periodontitis cohorts, supporting therapy-sensitive AO readouts in the oral compartment [99]. Separately (and importantly), systemic oxidative damage can be indexed by lipid peroxidation end-products: plasma MDA is significantly higher in periodontitis than in controls in clinical comparisons, supporting an increased systemic oxidative damage signal [100]. Finally, although many AO trials in chronic inflammatory diseases yield heterogeneous, null, or even harmful results, targeting oxidative stress/redox imbalance remains a plausible therapeutic direction when mechanistically grounded rather than implemented as indiscriminate supplementation [101].

#### 3.1.3. Hyperprolactinemia and Bone Resorption

Antipsychotics that antagonize dopamine D2 receptors (dopamine D2 receptors) frequently elicit hyperprolactinemia, with clinically meaningful between-compound and dose–response variability (e.g., paliperidone/risperidone showing prominent prolactin elevations, whereas aripiprazole generally tends to lower prolactin). This pattern supports hyperprolactinemia as a plausible mediator of downstream systemic morbidity, including adverse skeletal outcomes [7].

Mechanistically, elevated prolactin can dysregulate bone remodeling through perturbation of the receptor activator of nuclear factor κB ligand (RANKL)/osteoprotegerin (OPG) axis. Experimental hyperprolactinemia has been shown to increase the osteoblast-expressed RANKL/OPG ratio (RANKL ↑, OPG ↓), thereby favoring osteoclastogenesis and resorptive dominance [102].

Notably, cell context effects have been reported. For example, fetal osteoblast models have shown a decreased RANKL/OPG ratio under prolactin exposure [103], suggesting that prolactin’s net skeletal impact may depend on developmental/biological context and exposure state.

Clinically, this endocrine–skeletal vulnerability is consistent with meta-analytic evidence that SCZ is associated with lower bone mineral density (BMD) and increased fracture risk [104]. Medication-related falls and fractures risk is further shaped by psychotropic class effects (e.g., atypical antipsychotics ranking highest for falls, typical antipsychotics for fractures in a network meta-analysis) [105], and by longer-term fragility-fracture patterns discussed in clinical syntheses contrasting prolactin-raising versus prolactin-sparing agents [106].

This systemic osteoporotic tendency can synergize with localized alveolar bone destruction in periodontitis, where periodontal pathogens and host immune activation increase local RANKL signaling (notably from T and B cells and other sources) and shift the local RANKL/OPG balance, accelerating osteoclast-mediated resorption [107,108].

In patients with SCZ, the periodontal burden may be further compounded by antipsychotic exposure and associated hyperprolactinemia, which has been linked to periodontal risk and severity [109], and by broader oral health vulnerabilities described in older adults [110] and in case–control evidence linking PZ with poorer oral health indices [50]. The resulting shift toward bone resorption may therefore amplify CAL and reinforce the cumulative somatic burden in PZ, especially when real-world access barriers to dental care remain substantial in SMI populations [79].

### 3.2. Metabolic Comorbidities: The Inflammatory Catalysts

#### 3.2.1. The AGEs–RAGE Axis and Diabetes

In diabetes, sustained hyperglycaemia accelerates non-enzymatic glycation and the accumulation of AGEs, which can activate RAGE and amplify oxidative–inflammatory signaling. Beyond periodontal tissues, AGEs are also positioned as key mediators in cardiovascular disease pathophysiology and therapeutic targeting [111]. In periodontal tissues, this AGEs–RAGE signaling is a plausible mechanistic driver of enhanced tissue destruction and impaired repair observed in diabetes-associated periodontitis, alongside a broader cytokine-mediated inflammatory burden [20,33,34].

At the signaling level, AGEs–RAGE engagement promotes ROS generation, with experimental evidence supporting a key role for NADPH oxidase-dependent oxidative responses downstream of RAGE activation [60]. This AGEs–oxidative stress axis is also positioned as a central contributor to diabetic vascular injury, linking glycation burden to inflammatory and thrombogenic pathways [61]. Beyond membrane signaling, glycation can directly compromise intracellular protein function: in cardiomyocytes, glycation-related modifications affecting Ca^2+^ handling (including proteins such as sarco/endoplasmic reticulum Ca^2+^- adenosine triphosphate (ATP)ase (SERCA) and ryanodine receptors (RyR)) have been associated with disturbed calcium homeostasis and mechanical dysfunction in glycation stress states [112]. Counter-regulatory mechanisms include circulatings RAGE, which can function as a decoy receptor and potentially dampen ligand-driven RAGE activation [113]. Clinically, salivary and GFC AGEs/sRAGE profiles (together with IL-17) have shown discriminative potential across PD severity and glycaemic control strata, supporting the axis as both a mechanistic- and biomarker-level bridge between DM and PD [114].

#### 3.2.2. Endothelial Dysfunction and Cardiovascular Risk

Chronic periodontitis has been linked to a higher risk of ACVD, with epidemiological evidence suggesting an excess risk that persists after adjustment for conventional risk factors, while causality remains incompletely established [31,32,52]. Periodontal inflammation may contribute to endothelial dysfunction through systemic inflammatory and oxidative pathways that reduce NO bioavailability and impair vascular homeostasis [32,52,115]. In parallel, transient bacteremia and dissemination of periodontal pathobionts provide biological plausibility for vascular seeding and downstream pro-thrombotic and plaque-destabilizing effects, with *P. gingivalis* being a frequently discussed candidate pathogen [32,52,116,117].

In individuals with PZ, who frequently present a higher baseline cardiometabolic risk, periodontitis may represent an additional, potentially modifiable inflammatory burden within cardiovascular prevention strategies [2,4,118].

#### 3.2.3. Metabolic Syndrome

Patients with PZ, including SCZ, show a high prevalence of MetS, attributable to lifestyle factors and the iatrogenic metabolic burden associated with SGAs [2,4,85,86,87]. At the molecular–mechanistic level, MetS is underpinned by intertwined pathways linking adipose tissue dysfunction, insulin resistance, and inflammatory signaling [5]. Adipose tissue is an immuno-endocrine organ that actively modulates systemic metabolism and inflammatory tone [119]. In obesity-prone states and MetS, dysregulated adipose-derived mediators (adipokines) promote chronic, low-grade inflammation and contribute to insulin resistance and cardiovascular risk [77,120,121].

Mechanistically, immunometabolic reprogramming in adipose tissue is closely linked to macrophage metabolism; mitochondrial oxidative phosphorylation (OXPHOS) is a key determinant of tissue macrophage homeostasis and can shape inflammatory phenotypes relevant to cardiometabolic dysfunction [122]. In parallel, mitochondrial instability can amplify sterile inflammation: mtDNA leakage can act as a damage-associated molecular pattern (DAMPs), sustaining inflammatory signaling in cardiometabolic and age-related cardiovascular settings as well as in neurological disease contexts [17,28]. In psychiatric populations—where baseline cardiometabolic vulnerability is already elevated—this “metabolic–inflammatory” milieu may increase susceptibility to persistent infections and impaired resolution of chronic inflammatory foci, thereby plausibly reinforcing a bidirectional loop between systemic inflammation and periodontal breakdown [2,4,123,124].

#### 3.2.4. The TXNIP–NLRP3 Axis: The Metabolic Redox Sensor

A plausible mechanistic bridge between metabolic dysfunction and chronic inflammatory burden is the TXNIP–NLRP3 axis, which functions as a redox- and nutrient-sensitive inflammatory switch [125,126]. In PZ-spectrum populations—where oral disease burden appears increased—this axis may represent a convergence point between iatrogenic dysmetabolism and periodontal inflammation [33,50]. At the population level, SGA treatment is consistently linked to clinically relevant weight gain and adverse metabolic changes, including worsening glycaemic control, with substantial heterogeneity across individual agents [4,127,128]. In parallel, diabetes-related metabolic dysregulation is bidirectionally linked to periodontitis severity and inflammatory burden, supporting an oral–systemic loop relevant to metabolically vulnerable hosts [33,129]. Given the high cardiometabolic burden associated with SGAs, adjunctive metformin has a relatively strong evidence base for attenuating antipsychotic-associated weight gain and improving anthropometric/metabolic indices in SCZ-spectrum populations [130]. Placebo-controlled evidence synthesized in meta-analytic form supports clinically meaningful reductions in weight and BMI with metformin versus placebo, alongside improvements in insulin resistance measures [130], and randomized trials in SCZ/schizoaffective disorder further support this direction of effect [131]. Rather than asserting a uniform “periodontal tissue glucose rise” across all patients, a more conservative interpretation is that systemic hyperglycaemia and oxidative stress can create a permissive milieu for TXNIP upregulation and downstream inflammasome signaling.

At the molecular level, TXNIP has been reported to participate in inflammasome signaling, and high-glucose conditions can promote TXNIP–NLRP3 activation in experimental models [48,58]. In PD specifically, the NLRP3 inflammasome is increasingly described as a bidirectional driver of pathogenesis and tissue breakdown, supporting the relevance of this pathway to oral inflammatory burden [59]. Together, these data justify framing TXNIP–NLRP3 as a candidate “metabolic–redox” node that can amplify IL-1 family cytokine maturation (e.g., IL-1β and IL-18) and sustain low-grade inflammation when persistently activated. Crucially, oral pathogens may extend this inflammatory signal beyond the periphery: *P. gingivalis* has been shown to trigger microglial activation and neurodegenerative processes through NOX4-dependent mechanisms [11]. This convergence supports a biologically plausible oral–systemic–brain relay in which oxidative stress pathways (including NOX signaling and AGEs–RAGE-related redox amplification) could facilitate neuroinflammatory feedback loops in susceptible hosts [11,61,112,132]. This integrated oral–systemic–brain pathway, converging on the TXNIP–NLRP3 node, is summarized in Figure 1.

### 3.3. PD as a “Bio-Oxidative Pump”

PD may function as a chronic “bio-oxidative pump” by sustaining persistent local inflammation with systemic spillover of inflammatory mediators and microbe-derived products, thereby contributing to low-grade inflammation in vulnerable populations (e.g., SMI) [2,8,23]. Within this framework, oxidative stress generated during chronic periodontal inflammation can aggravate systemic redox imbalance, and periodontitis has been associated with altered circulating oxidativestress biomarkers [13,133]. Excessive ROS can compromise mitochondrial bioenergetic homeostasis and thereby amplify oxidative distress [134,135]. Importantly, clinical evidence also supports a link between periodontal inflammation and mitochondria-related oxidative stress, as intensive periodontal treatment in patients with type 2 DM (T2DM) and periodontitis has been associated with reduced mitochondrial reactive oxygen species (mtROS) alongside improved endothelial function and metabolic control [136].

Mitochondrial respiratory chain supercomplexes (SCs) are increasingly recognized as structural/functional assemblies that can improve electron transfer efficiency and modulate ROS generation, making SC organization relevant for preserving mitochondrial performance under stress [137,138]. Experimental work further supports the concept that respiratory supercomplex association can limit ROS production from complex I, whereas disruption of SC organization enhances O_2_^•−^ generation—consistent with a self-reinforcing cycle linking mitochondrial inefficiency and redox imbalance [139].

#### 3.3.1. Mitochondrial Dynamics, Disrupted Mitophagy, and Microglial Activation

Chronic PD sustains a persistent inflammatory and oxidative milieu that can disrupt mitochondrial homeostasis. Under chronic stress, mitochondrial quality control processes (including mitochondrial dynamics and mitophagy—the selective clearance of damaged mitochondria) may become insufficient, favoring the accumulation of dysfunctional organelles and the propagation of sterile inflammatory signals [140,141]. In diabetic–periodontitis contexts, mitochondrial dysfunction is increasingly emphasized as a mechanistic amplifier of periodontal pathology, supporting the plausibility of impaired mitochondrial quality control in chronic periodontal inflammation [142].

When mitochondrial quality control fails, mtDNA leakage can occur through membrane permeabilization pathways (including mitochondrial permeability transition pore (mPTP)-associated routes) and other export mechanisms; leaked mtDNA can behave as a DAMPs and be recognized by innate immune sensors, including cGAS–STING, TLR9, and inflammasomes, thereby sustaining and amplifying sterile inflammation [28,143]. Because barrier integrity and neuroimmune setpoints may be altered during chronic systemic inflammation, a more conservative and defensible formulation is that peripheral DAMPs and redox signaling can contribute to neuroinflammatory priming rather than positing that mtDNA crosses the BBB as a universal mechanism [144].

In parallel, PD is increasingly framed as bidirectionally linked to NLRP3 inflammasome activity, strengthening the rationale for an oral-to-systemic inflammasome relay [59]. At the periodontal level, oxidative imbalance is measurable, with altered AO/oxidative profiles reported in chronic periodontitis and supported by synthesis evidence for systemic oxidative stress biomarker alterations [13,133]. Conceptually, it is also important to distinguish oxidative distress (damage-associated) from oxidative eustress (signaling), as chronic inflammatory states tend to shift the balance toward distress [134].

Beyond systemic priming, periodontal pathogens may provide a brain-directed inflammatory “spark.” Notably, *P. gingivalis* has been shown to trigger microglial activation and neurodegenerative processes through NOX4-dependent mechanisms, offering a plausible oral–brain link via redox-driven neuroinflammation [11]. Population-based evidence also supports clinically relevant co-occurrence patterns between PD and SCZ (including effects related to antipsychotic exposure), reinforcing the need to consider oral inflammation as part of the broader iatrogenic/metabolic vulnerability profile in PZ-spectrum disorders [89].

#### 3.3.2. The Oral–Brain Axis: Beyond the “Bio-Oxidative Pump”

PD can sustain a chronic peripheral inflammatory state because the periodontal niche becomes a self-reinforcing dysbiotic ecosystem that subverts host immunity and impairs resolution. In molecular terms, persistent dysbiosis continuously supplies PAMP exposure (e.g., LPS and other microbial motifs) that engages systemic innate immune circuits (pattern recognition receptor (PRR)-driven inflammatory programs), thereby producing a durable “input signal” to peripheral innate immunity [21,23,30]. Within periodontal tissues, hypoxia and repeated PAMP exposure (notably LPS) provide a permissive context for inflammasome-related pathways. Specifically, *P. gingivalis* LPS can synergize with hypoxic stress to support caspase-1-linked inflammatory processing [145], while NLRP3 activity is increasingly recognized as bidirectionally coupled to periodontal pathogenesis—both contributing to tissue-destructive inflammation and being reinforced by ongoing periodontal inflammatory signaling [59]. A key escalation step is the conversion of local oral inflammation into repeated systemic exposures, which is mechanistically important because repeated PAMP encounters can progressively increase the probability of innate priming. Daily oral activities (e.g., toothbrushing) can generate transient bacteremia, and meta-analytic evidence indicates that plaque burden and gingival inflammation increase bacteremia prevalence after toothbrushing [146]. Controlled clinical data further show that toothbrushing can produce measurable bacteremia with oral species [147]. Even when each episode is short, their recurrence can function as a cumulative peripheral inflammatory “training set,” plausibly lowering the threshold for downstream neuroimmune amplification [146,147]. For peripheral signals to meaningfully amplify neuroinflammation, barrier mechanisms become central, and the BBB is best conceptualized as a regulated endothelial interface where permeability can shift via specific molecular routes. Experimental work indicates that *P. gingivalis* bacteremia can increase BBB permeability via a mechanism consistent with transcytosis remodeling—notably the major facilitated superfamily domain-containing 2A (Mfsd2a)/Caveolin-1 axis, which points to caveolae-associated vesicular transport as a key permeability lever [63]. In a murine model of *P. gingivalis*-induced periodontitis, BBB dysfunction was accompanied by pathogen/immune cell infiltration and glial activation, together with neuronal injury markers and cognitive impairment—supporting “brain barrier dysfunction” as a functional bridge between oral infection and central nervous system (CNS) pathology [64]. In parallel, systemic metabolic/redox stress can supply DAMPs that synergize with PAMPs at the level of innate immune sensing. mtDNA release during mitochondrial quality control failure behaves as a DAMPs and can engage innate immune sensors (including cGAS–STING, TLR9, and NLRP3), reinforcing sterile inflammation and plausibly biasing CNS immune tone when BBB integrity is reduced [28]. This matters mechanistically because it provides a route for sustained inflammation even when microbial exposure varies, through continued endogenous danger signaling. Within the CNS, microglia represent the principal cellular “gain control” that converts peripheral danger signals into sustained neuroinflammation, and a key molecular amplifier here is redox–inflammatory coupling. Mechanistically, *P. gingivalis* can trigger microglial activation and neurodegenerative processes through NOX4-dependent pathways, with NOX4-linked ROS acting as a redox amplifier of inflammatory signaling [11]. Mitochondrial vulnerability under sustained oxidative stress, while extensively studied in non-psychiatric contexts (e.g., drug-tolerant cancer persister states), is invoked here only as a conceptual analogy and not as a shared disease mechanism.

Together, these data support a feed-forward loop in which (i) recurrent oral translocation/bacteremia (PAMP load), (ii) systemic DAMPs burden (e.g., mtDNA-linked innate sensing), (iii) BBB vulnerability/permeabilization (including transcytosis remodeling), and (iv) microglial NOX4–ROS amplification converge to stabilize neuroinflammation even when the initiating oral stimulus fluctuates [11,28,63,64].

From a psychiatric standpoint, this microglia-centered model is clinically relevant because people with PZ are disproportionately exposed to cardiometabolic vulnerability and treatment-related dysmetabolism—factors that elevate peripheral inflammatory tone and can sustain a background immunometabolic burden [2,4]. Population-based evidence further links SCZ and antipsychotic exposure to poorer periodontal outcomes (including medication-related salivary dysfunction), which is consistent with bidirectional amplification between chronic oral inflammation and systemic/iatrogenic risk [89]. In this setting, the concept of microglial priming by systemic inflammatory stimuli—and the broader integration of neuroinflammation with oxidative stress in SCZ—provides a coherent framework for how peripheral inflammatory load could translate into heightened neuroimmune “background noise,” as well as for emerging adjunctive strategies that target microbiome–inflammation pathways (psychobiotics) and optimize interventions using nanotechnology and artificial intelligence [148]. Crucially, and specifically for the redox amplification mechanism, experimental evidence shows that *P. gingivalis* can trigger microglial activation and neurodegenerative processes through NOX4-dependent signaling, with NOX4-linked ROS acting as a proximate amplifier of inflammatory outputs—supporting a mechanistic route by which recurrent peripheral oral challenges could lower the threshold for sustained neuroinflammation in susceptible individuals [11].

## 4. Clinical Implications

The convergence model advanced in this review conceptualizes excess somatic morbidity in PZ as an emergent consequence of interacting iatrogenic, metabolic, and oxidative–inflammatory processes. Rather than operating in parallel, these domains can form a biologically self-reinforcing loop in which antipsychotic exposure and baseline cardiometabolic vulnerability increase inflammatory tone, while chronic oral inflammation adds a persistent peripheral “input” that can further amplify systemic risk and functional decline [2,4,23,50]. This integrative framing is aligned with the consensus view that periodontitis is independently associated with major non-communicable diseases, supporting a shift from purely local periodontal management toward coordinated medical–dental prevention strategies [149].

In this context, SGAs can be clinically relevant because they may perturb both oral and systemic homeostasis early in treatment. On the oral side, psychotropic medication burden is associated with xerostomia and salivary dysfunction, which compromises saliva’s protective functions (mechanical clearance, buffering, and antimicrobial defense), thereby facilitating dysbiotic biofilm persistence and periodontal vulnerability [150,151,152,153]. On the systemic side, antipsychotic treatment is linked to metabolic adverse outcomes in SCZ, including dysglycaemia and insulin resistance, creating a permissive substrate for downstream inflammatory cascades [81,86].

Mechanistically, metabolic dysregulation and hyperglycaemia-related biology can converge on periodontal tissue susceptibility through inflammatory amplification pathways that include cytokine signaling and AGEs-mediated inflammatory stress. The periodontitis–diabetes relationship is biologically plausible and clinically relevant, with cytokine networks (e.g., IL-1β, TNF-α, IL-6) and the AGEs–RAGE axis contributing to oxidative stress, impaired repair, and accelerated periodontal destruction [20,33,61]. Once established, periodontitis can behave as a chronic systemic inflammatory amplifier, sustaining systemic spillover of inflammatory mediators and oxidative stress signatures, including reduced circulating TAC [13,133]. In parallel, inflammasome activity (including NLRP3) is implicated in periodontal pathogenesis, and hypoxia–LPS synergy can potentiate caspase-1-dependent inflammatory activation in periodontal contexts [59,145].

Clinically, this low-grade inflammatory state can worsen cardiometabolic risk and may also facilitate neuroimmune amplification. Frequent transient bacteremia from routine oral activities has been documented, and experimental work supports BBB vulnerability under *P. gingivalis* bacteremia via Mfsd2a/Caveolin-1-mediated transcytosis, while microglial activation can be propagated through redox mechanisms such as NOX4-dependent pathways [11,63,146,147]. In addition, inflammatory “memory” mechanisms may help explain why repeated peripheral inflammatory exposures can have disproportionate downstream effects in susceptible hosts [21,30]. Epidemiologically, population-based data support clinical attention to PD in SMI, including SCZ cohorts treated with antipsychotics [37,89].

### 4.1. The Pathological Cascade: From Iatrogenesis to Neurobiological Feedback

#### 4.1.1. Molecular Dissemination: The NLRP3 and AGEs–RAGE Axis

The periodontium can become a clinically “silent” yet biologically active source of chronic inflammation that contributes to systemic inflammatory burden, a mechanistic framework consistent with periodontal links to cardiovascular and other inflammatory comorbidities [8,22,23]. In patients exposed to metabolic derangements—particularly chronic hyperglycemia and metabolic syndrome risk phenotypes that are prevalent in severe psychiatric illness and can be exacerbated by psychotropic medication—systemic priming may further amplify periodontal inflammation and its systemic spillover [2,33,154].

At the molecular level, NLRP3 inflammasome involvement in PD pathogenesis supports the plausibility of enhanced maturation and release of key pro-inflammatory cytokines such as IL-1β and IL-18 in periodontal inflammation [59]. Hyperglycemia-associated signaling can intersect with this inflammatory circuitry; for example, high glucose-induced TXNIP–NLRP3 activation has been reported in non-periodontal tissue contexts [48] and is mechanistically consistent with ROS-sensitive TXNIP–NLRP3 coupling described in core inflammasome biology [125,155]. In parallel, the AGEs–RAGE axis can sustain a self-perpetuating oxidative stress loop, with direct mechanistic evidence that AGEs–RAGE engagement activates NADPH oxidase-dependent ROS generation [60,61,156]. Although not periodontal-specific, redox modulation by biomolecular condensate interfaces provides a plausible amplification principle for local redox signaling and inflammatory persistence [157].

Collectively, these mechanisms align with the concept that chronic periodontal inflammation can contribute to systemic inflammation and comorbidity risk through sustained innate immune activation, oxidative stress, and inflammatory “memory” phenomena [6,30,158].

#### 4.1.2. The Neurobiological “Second Hit”

Periodontitis should be conceptualized as a chronic inflammatory focus capable of sustaining a persistent systemic burden of pro-inflammatory and pro-oxidant mediators through episodic bacterial translocation and the “spill-over” of cytokines/chemokines and microbial products from the periodontal lesion into the circulation [8,21]. In this framework, peripheral inflammation is not merely a clinical epiphenomenon but a repetitive stimulus that can “prime” the endothelium, immunometabolic axes, and systemic oxidative responses, thereby increasing the likelihood that a subsequent event (hit) will exert disproportionate effects on neurovascular and neuroimmune compartments [23].

From a microbiological standpoint, periodontitis is increasingly framed as a polymicrobial dysbiosis rather than a single-pathogen infection. In this ecology, *P. gingivalis* may act as a low-abundance keystone that reshapes community structure, yet tissue destruction is better interpreted as a community-level phenotype involving additional taxa (e.g., red-complex organisms and other pathobionts), consistent with keystone/PSD (polymicrobial synergy and dysbiosis) concepts [21,159,160]. Clinically, SMI is associated with higher prevalence/severity of PD, supporting oral inflammation as a plausible and modifiable contributor to systemic burden [161]. For cardiometabolic comorbidity, a systematic review/meta-analysis reports frequent detection of periodontal bacterial DNA in coronary atheromatous plaques, consistent with dissemination pathways, while complementary reviews summarize atherogenic mechanisms for diverse periodontal taxa [48,162]. Collectively, these data support a polymicrobial framing for oral–systemic hypotheses in PZ and cardiometabolic disease rather than a *P. gingivalis*-only narrative [163,164].

In SCZ/PZ, converging evidence supports an intrinsic vulnerability of redox homeostasis, in which reduced glutathione (GSH) deficits/instability and impaired AO defenses may reduce the biological “reserve” required to buffer peripherally induced oxidative stress [9]. This vulnerability is neurodevelopmentally relevant: experimental models with GSH deficit during development produce alterations in parvalbumin-positive γ-aminobutyric acid (GABA) interneurons—a critical node for cortical network synchronization and for cognitive/psychotic phenotypes [165]. In addition, a 7 Tesla proton magnetic resonance spectroscopy (7T ^1H-MRS) meta-analysis reports significantly lower GSH in PZ compared with controls, supporting the notion that cerebral AO capacity can be compromised before peripheral stressors reach a pathogenic threshold [62]. Accordingly, the same peripheral inflammatory oxidative signaling that may be tolerable under intact redox capacity can operate as a “second hit” in the context of reduced GSH and the glutamatergic/GABAergic disturbances described in SCZ [166].

A key mechanism that renders translation from peripheral oral inflammation to neuroinflammation biologically plausible is the disruption of the BBB. In a *P. gingivalis* bacteremia model, increased BBB permeability has been reported via a transcytosis-dominant mechanism (the Mfsd2a/Caveolin-1 axis), suggesting a gateway for peripheral mediators—and potentially microbial components—to access the brain compartment [63]. Once microglial activation is initiated, oxidative amplification becomes a central accelerator of pathology: *P. gingivalis* (particularly LPS) has been associated with the induction of ROS and a pro-inflammatory response (IL-6/IL-8) via NOX4 in microglia, while microglia-conditioned media reduced neuronal viability and increased tau markers in a NOX4-dependent manner [11]. Conceptually, the proposed neurobiological vulnerability sequence can be summarized as periodontitis → systemic inflammation/ROS → increased BBB permeability → microglia (NOX4/NOX) → sustained central oxidative stress → synaptic dysfunction and cognitive vulnerability, while acknowledging that current evidence strongly supports mechanistic plausibility but does not yet establish definitive clinical causality across all stages [11,63].

To operationalize this model empirically—particularly in clinical/translational studies—an oxidative stress biomarker strategy that captures both systemic and oral compartments is useful. F2-IsoPs are widely regarded as robust in vivo indices of lipid peroxidation [167] and can be measured, for example, as urinary 8-iso-PGF2α; in parallel, soluble NOX2-derived peptide (sNOX2-dp) can serve as a marker of NOX2 activation [168]. In the oral compartment, salivary 8-OHdG has been synthesized meta-analytically as an oxidative DNA damage marker in PD, showing significant differences between patients and healthy controls [169]. Taken together, these readouts enable direct testing of whether periodontal inflammatory activity and NOX activation/lipid peroxidation track with neuropsychiatric vulnerability in a compounded vulnerability framework [167,168,169].

Finally, the shift from transient oxidative signaling (“eustress”) to persistent pathological oxidation (“distress”) provides a unifying language for chronicity. A conceptual synthesis of redox regulation explicitly distinguishes adaptive oxidative signaling from cumulative pathological oxidation [51], and within the Kelch-like ECH-associated protein 1 (KEAP1)–nuclear factor erythroid 2-related factor 2 (NRF2) axis, thiol sensing (KEAP1 cysteines) constitutes a major control point for initiating AO programs [170]. SCZ-relevant evidence further links perturbation of KEAP1–NRF2/heme oxygenase-1 (HO-1) signaling to oxidative stress/ferroptosis phenotypes and neuronal injury [7]. Thus, under sustained peripheral inflammatory oxidative load (e.g., active periodontitis), failure of KEAP1–NRF2 control may facilitate the conversion of transient redox stress into self-perpetuating oxidative neuroinflammation, consistent with a compounded vulnerability paradigm [51,171]. Importantly, recent evidence suggests that redox-sensitive transcription factors (including nuclear factor κ-B (NF-κB) and NRF2) function as convergent nodes linking oxidative distress to sustained inflammatory signaling, providing a rationale for polyphenol-based modulators in low-grade systemic inflammatory phenotypes [172].

### 4.2. Multidimensional Interventions: Breaking the Loop

The conceptual framework of this model can be understood as a progression through four interdependent stages, transforming psychiatric vulnerability into systemic somatic and neurological decline [2,4]. This sequence converts pharmacological risk into cumulative structural damage through oxidative stress and progressive depletion of AO buffering capacity, with periodontal inflammation acting as an additional upstream amplifier of systemic redox burden [2,4,13,173]. Table 2 synthesizes the proposed convergence cascade, illustrating how metabolic iatrogenesis and the oral AGEs–RAGE axis can prime systemic amplification, including mtDNA danger signaling and inflammasome-linked pathways, thereby increasing the likelihood of downstream neurovascular and neuroimmune consequences [28,59]. In periodontitis-focused synthesis work, periodontal microbiota-driven inflammasome activation is explicitly framed as a mechanism that can propagate tissue inflammation via IL-1β/IL-18 and DAMPs signaling, supporting the biological plausibility of an oral-to-systemic inflammatory contribution within this cascade [59].

To mitigate the systemic decline associated with this convergence, a tripartite approach is typically proposed as follows: (i) periodontal infection control, (ii) cardiometabolic risk management, and (iii) integrated service delivery tailored to SMI populations [17,33,41]. A cornerstone of periodontal control is SRP, which targets the upstream driver—subgingival dysbiosis—and can reduce systemic inflammatory burden in parallel with local clinical improvement [33,41,53]. Evidence synthesis indicates that NSPT can reduce systemic CRP, supporting the concept that treating periodontal inflammation may lower systemic inflammatory “noise” [53]. In addition, periodontal therapy appears to modify oral fluid inflammatory profiles: in clinical gingival crevicular fluid (GCF) monitoring, IL-1β and IL-18 can decrease toward healthy levels following nonsurgical periodontal treatment, while upstream inflammasome-related markers (e.g., NLRP3/caspase-1) may remain elevated, consistent with residual inflammatory activity and reinforcing the need for multi-marker interpretation over single readouts [69].

Beyond CRP, metabolic endpoints have also been examined in evidence syntheses, particularly in diabetes-focused analyses. In people with diabetes, a major Cochrane review reports a moderate-certainty absolute HbA1c reduction of 0.43% at 3–4 months after periodontal treatment [55]. This finding supports using periodontal care as an adjunct within broader metabolic management rather than as a standalone glycemic intervention [33,55]. Complementary umbrella-level syntheses similarly report that periodontal therapy may improve glycaemic control, reinforcing bidirectional periodontal–metabolic links [174].

Pharmacological strategies can be used to manage antipsychotic-associated weight gain and cardiometabolic risk within evidence-based clinical frameworks; for example, professional guidance documents synthesize options used in routine care [4]. Given the centrality of oxidative stress and glutathione-related mechanisms in PZ biology [175], redox-informed adjunct concepts often focus on restoring AO capacity and modulating NRF2–KEAP1-linked stress responses [7,166,170]. N-acetylcysteine (NAC), as a glutathione precursor with additional glutamatergic and immunomodulatory effects, has been evaluated as an adjunctive treatment in SCZ. A meta-analysis of randomized controlled trials suggests improvements in total and negative symptom severity after longer treatment durations (e.g., 24 weeks), with signals for cognitive outcomes [176]. Consistent with this, trial-level evidence reports working memory benefits with adjunctive NAC in PZ-spectrum populations [177]. While these findings require replication and careful phenotypic stratification, they support NAC as a plausible redox-targeted adjunct within an integrated periodontal–metabolic framework [176,177].

Metabolic-state heterogeneity may also influence responsiveness to redox- or mitochondria-relevant interventions; multivariate analyses in oncology illustrate that vulnerabilities can cluster by metabolic state, motivating a precision medicine mindset when translating redox concepts across diseases [178]. For longitudinal evaluation, panels that combine oxidative lipid damage (F2-IsoPs) and oxidative DNA damage (8-OHdG) can provide complementary information on systemic vs. local oxidative injury [167,169]. Finally, mechanistic bridges to neuroinflammation can be anchored in evidence that periodontal pathogens can trigger microglial activation via NOX-related oxidative pathways, supporting the plausibility (not proof) of an oral–systemic–brain inflammatory loop [11,23]. Converging neuropsychiatric synthesis work further frames NLRP3 as a stress-linked neuroinflammatory pathway and explicitly includes SCZ among conditions where NLRP3-modulating strategies are being considered, providing a rationale for future stratified trials that test whether reducing periodontal inflammasome-related “noise” can improve downstream inflammatory phenotypes in vulnerable psychiatric subgroups [179].

### 4.3. Future Research Directions

#### 4.3.1. Toward Evidence-Based Redox Interventions

Future work should prioritize adequately powered interventional trials testing whether improving periodontal inflammation can reduce systemic inflammatory and redox burden in people with PZ, while also tracking clinically meaningful metabolic and vascular endpoints. In addition to PZ-focused outcomes, trial architecture should explicitly incorporate validated periodontal-to-metabolic and periodontal-to-inflammatory endpoint frameworks (e.g., redox and glycaemic measures), as exemplified in diabetes–periodontitis intervention studies and evidence syntheses [53,55,180].

A key mechanistic objective is to validate redox pathways using interpretable biomarker panels (rather than single markers), including AO status in saliva and serum alongside inflammation-linked oxidative stress markers, measured before and after periodontal treatment [13,181].

Beyond short-term oxidative stress readouts, trials should incorporate long-term metabolic follow-ups to test whether periodontal inflammation control can serve as a non-pharmacological adjunct for glycaemic improvement. In people with diabetes, the most robust synthesis in the provided bibliography supports a short-term HbA1c reduction after periodontal therapy, reinforcing the rationale for periodontal care as an adjunct within broader metabolic management [33,55].

#### 4.3.2. Methodological Appraisal: Challenges in Measuring the Redox Load

Many studies still rely on “static” damage markers (e.g., MDA, TAC), which primarily reflect accumulated oxidative injury rather than real-time redox flux. This limits causal inference and makes the timing of sampling critical [41,167,182].

Another limitation is that peripheral measurements (blood/saliva) represent systemic averages and may dilute tissue- or niche-specific signals relevant to periodontal lesions or neurobiological processes. Therefore, future designs should combine systemic markers with oral-site readouts (e.g., saliva and GCF) and interpret them as integrated panels [39,59,70,91].

For higher specificity, promising approaches include pairing lipid peroxidation markers (F2-IsoPs) with oxidative DNA damage (8-OHdG) to capture complementary aspects of oxidative stress biology [167,169].

#### 4.3.3. Beyond Standard Therapy

Inflammasome-related pathways (including NLRP3) and broader immunometabolic drivers are increasingly discussed in periodontal pathogenesis and remain plausible nodes within oral–systemic inflammatory spillover hypotheses [59]. Experimental work in periodontal ligament fibroblasts exposed to *P. gingivalis* LPS supports a ROS–TXNIP–NLRP3 axis as an initiating mechanism for downstream inflammatory responses, providing a mechanistic bridge between redox sensing and inflammasome activation [183]. In parallel, a reverse-translational redox framework in a redox-vulnerable mouse model (glutamate-cysteine ligase modifier subunit knockout (*Gclm*-KO) ± GBR 12909 (GBR)) shows that mitochondrial oxidative stress can propagate microcircuit-relevant dysfunction and that the mitochondria-targeted antioxidant mitoquinone mesylate (MitoQ) can normalize oxidative stress-linked molecular readouts, including mitophagy markers and parvalbumin interneuron (PVI)-related mitochondrial signatures [67]. A recent narrative review further consolidates the view that periodontal microbiota-driven NLRP3 activation can promote IL-1β/IL-18 release and DAMPs signaling in periodontitis [59].

From a translational standpoint, these data motivate—but do not yet justify—adjunctive approaches beyond SRP that are explicitly mechanism guided and biomarker anchored. One candidate direction is upstream TXNIP modulation: in human periodontal ligament cells, hypoxia-induced ROS activated the TXNIP–NLRP3 axis, and N-acetylcysteine (NAC)-mediated ROS reduction downregulated TXNIP expression while inhibiting NLRP3-related factors and attenuating inflammatory outputs [58]. Mechanistic evidence from non-periodontal metabolic stress models indicates that sirtuin 1 (SIRT1) can function as an upstream negative regulator of TXNIP–NLRP3 activation, supporting the plausibility of “SIRT1-linked” strategies as an upstream approach to TXNIP–NLRP3 modulation that warrants periodontal-targeted validation before clinical extrapolation [48].

MitoQ can be positioned as a candidate adjunct specifically for mechanistically defined subgroups rather than as generic supplementation. In a reverse-translational framework, circulating exosomal microRNA-137 (*miR*-137) and cytochrome c oxidase subunit 6A2 (COX6A2), together with mitophagy marker alterations, were proposed as a biomarker set that may stratify early-PZ patients into mitochondrial dysfunction versus non-dysfunction profiles, thereby enabling biomarker-guided trial designs [67]. Importantly, clinical efficacy of MitoQ in PZ remains to be established, but feasibility for translational testing is supported by the existence of a Yale Medicine clinical trial listing exploring MitoQ in early SCZ-spectrum disorders [68].

Overall, a “personalized/precise oxidative stress therapy (POST)-like” framework argues that adjunctive redox interventions should be phenotype- and biomarker-defined, and should prioritize mechanistic endpoints (redox balance, mitophagy, inflammasome signaling, and endothelial function) over indiscriminate AO use. This caution is supported by redox biology evidence emphasizing that ROS also function as signaling molecules (not only toxic byproducts), such that excessive, non-targeted AO suppression may disrupt physiological signaling and lead to paradoxical effects [184]. In addition, evidence-based discussions of oxidative/antioxidative balance highlight that both oxidative stress and “antioxidative stress” can be detrimental, motivating measurement-informed and individualized supplementation rather than blanket approaches [65].

### 4.4. Limitations and Critical Appraisal

Although associations between periodontitis, cardiometabolic dysregulation, and systemic inflammation in SMI are consistently reported, the current evidence base remains predominantly observational, leaving causality and directionality insufficiently established [15,21,23,36,161]. Mendelian randomization analyses do not currently support a bidirectional genetic causal relationship between periodontitis and major psychiatric disorders [48], reinforcing the interpretation that the observed associations may largely reflect acquired pathways rather than shared genomic predisposition. Interpretation is further constrained by substantial heterogeneity in SMI phenotypes, antipsychotic exposure, smoking/substance use, SES, and access to dental care, which increases residual confounding and limits cross-study comparability [4,15,16,66,123]. Throughout this review, we use psychosis (clinical syndrome), schizophrenia (DSM-5 disorder), schizophrenia-spectrum disorders (SSD), and severe mental illness (SMI) as a broader umbrella; these populations are not interchangeable and differ in chronicity, medication exposure, and somatic comorbidity profiles. Residual confounding by smoking, alcohol and substance use, socioeconomic status, antipsychotic exposure (type, dose, duration), metabolic syndrome, oral hygiene access, and multimorbidity is therefore substantial and may attenuate, mediate, or partially explain the periodontitis–psychosis association.

Common low-grade systemic inflammation readouts (e.g., CRP/hs-CRP, NLR, IL-6) are pragmatic for risk stratification but lack specificity; interpreted in isolation, they may lead to over-interpretation [185,186,187]. This observation supports a panel-based approach and pre-specified multi-marker interpretation frameworks rather than single-marker inference [8,53,70]. Biomarker heterogeneity across studies (assay platforms, sampling matrices, pre-analytical handling) further restricts cross-study comparability, particularly for emerging redox markers (sNOX2-dp, F2-IsoPs, 8-OHdG, MDA, and GSH/GSSG) and for salivary or oral fluid analytes (e.g., salivary NLRP3). Periodontal phenotyping itself is heterogeneous (clinical attachment level, bleeding on probing, radiographic bone loss, contemporary periodontal staging and grading classification vs. self-report), and psychiatric phenotyping ranges from first-episode psychosis cohorts through DSM-defined schizophrenia to broader SMI registries—adding a layer of definitional variability that should be considered when integrating findings. Mechanistic models centered on redox imbalance and the TXNIP–NLRP3 axis are biologically coherent yet likely non-exclusive; competing and intersecting pathways (e.g., endothelial dysfunction, mitochondrial dysfunction/DAMPs signaling, and neuroimmune activation) should be integrated and tested in longitudinal designs [11,28,59,140,181]. Several specific translational gaps deserve explicit acknowledgement. First, there is currently no causal clinical evidence that treating periodontitis modifies psychiatric trajectories in PZ-spectrum populations. Second, mechanistic pathways such as NOX4-mediated microglial activation, mtDNA leakage as a DAMP, and TXNIP–NLRP3 convergence are largely supported by preclinical models or by extrapolation from non-psychiatric tissue contexts; direct human confirmation in PZ remains pending. Third, integrated oral–psychiatric care pathways face structural obstacles (funding, multidisciplinary coordination, trial-design heterogeneity) that have, to date, limited large-scale interventional studies. Fourth, the OXPHOS vulnerability concept previously discussed in the cancer persister literature is retained here only as a conceptual analogy and not as a shared disease mechanism.

## 5. Conclusions

This review proposes a tentative “pathological confluence” model in which SMI, antipsychotic-related metabolic vulnerability, and periodontal dysbiosis may plausibly interact to sustain a self-reinforcing cycle of oxidative stress, chronic low-grade inflammation, and hypothesized downstream vascular and neuroimmune consequences. Rather than viewing PD as a peripheral comorbidity, the model frames the periodontium as a candidate upstream amplifier of systemic redox and inflammatory burden, especially in contexts where self-care constraints and cardiometabolic risk trajectories already bias patients toward chronic immune activation. This framing is mechanistic and associative; it does not imply established causality in psychosis populations.

Mechanistically, the convergence of mitochondrial stress signals with inflammasome-linked pathways provides a coherent (though non-exclusive) explanatory layer: contemporary periodontal synthesis work highlights that NLRP3 activation in response to periodontal microbiota can amplify disease progression through IL-1β/IL-18 and DAMPs-associated signaling [59]. In parallel, emerging SCZ-focused mechanistic data support NLRP3 as a candidate neuroimmune node in an inflammatory subtype of disease, with astroglial NLRP3-dependent inflammatory phenotypes that are reversible upon targeted inhibition in preclinical models [188]. Together, these lines of evidence justify a cautious and explicitly hypothesis-driven oral–systemic–brain framework that requires rigorous longitudinal and interventional study designs before any translational claim can be made.

Clinically, and in the absence of direct interventional evidence in psychosis populations, the model should be interpreted as a research-oriented framework rather than as a basis for current treatment recommendations. It motivates a multidimensional research agenda built around the (i) evaluation of proactive periodontal infection control and maintenance as a candidate modifiable factor, (ii) assessment of early and sustained cardiometabolic risk management during high-leverage phases of antipsychotic exposure, and (iii) exploration of integrated service delivery pathways that could potentially reduce access barriers and fragmentation of care in SMI populations. From a measurement standpoint, this review argues against single-marker interpretations and favors panel-based monitoring. Recent clinical evidence in periodontal but not psychiatric cohorts also suggests that oral fluid markers related to inflammasome activity could become pragmatically useful: salivary NLRP3 is reported to be elevated in chronic periodontitis [189], and GCF profiles change after nonsurgical periodontal therapy in patterns consistent with partial resolution and residual inflammatory activity [69]. However, none of these biomarkers have yet been validated in psychosis populations, and these signals are hypothesis generating only and should be validated with standardized protocols, harmonized thresholds, and prespecified analytic plans before any clinical inference.

Future work should therefore prioritize the following: (1) prospective cohorts that jointly phenotype oral status, metabolic trajectories, and inflammatory/redox panels in SMI; (2) adequately powered randomized or pragmatic trials testing whether periodontal therapy and adjunctive lifestyle/metabolic interventions produce clinically meaningful downstream effects in psychosis populations, where such evidence does not currently exist; and (3) mechanistic studies integrating mitochondrial danger signaling, endothelial dysfunction, and neuroimmune activation to clarify which pathways are causal, which are epiphenomenal, and which subgroups might benefit from targeted interventions. Overall, the convergence framework provides a structured research agenda for moving from association toward future precision-oriented prevention in a population with substantial unmet physical health needs. Until prospective and interventional human evidence specifically in psychosis populations becomes available, the framework outlined here should be regarded as a hypothesis-generating synthesis intended to guide research and integrated care planning, and not as a confirmed causal model or as a basis for current periodontal interventions in patients with severe mental illness.

## Figures and Tables

**Table 2:** Summary of the pathological cascade.

Stage	Drivers	Key Pathways	Consequences	Readouts/Interventions (By Evidence Tier)
I. Iatrogenic–metabolic initiation	SGAs; weight gain; insulin resistance/hyperglycemia; xerostomia; hyperprolactinemia	PI3K/AKT/mTOR dysregulation; advanced glycation end-products (AGEs) formation; redox shift; RANKL/OPG imbalance	Increased cardiometabolic risk; impaired oral defensive capacity; increased susceptibility to periodontitis	Clinically established: BMI trajectory, fasting glucose/HbA1c, lipid profile, salivary flow/pH, prolactin. Research/exploratory: preclinical redox markers in saliva; metabolic trajectory stratification (e.g., LBRI subgroup analyses). Therapeutic perspectives: early metabolic monitoring during high-leverage SGAs exposure; panel-based interpretation rather than single-marker inference [2,4,39].
II. Oral dysbiosis (“bio-oxidative pump”)	Periodontal pocket biofilm; hygiene/access barriers; smoking/substance use	TLR-driven inflammatory priming and NF-κB signaling; neutrophil oxidative burst; ROS/RNS excess; AO depletion (effect sizes vary by cohort/context)	Local tissue destruction + systemic inflammatory spillover; systemic AO drain	Clinically established: probing depth, clinical attachment level (CAL), bleeding on probing (BOP). Research/exploratory: GCF/saliva IL-1β/IL-6/TNF-α, MMPs, oxidative stress markers, and salivary NLRP3 as an emerging oral fluid inflammasome readout. Therapeutic perspectives: nonsurgical periodontal therapy (NSPT) with longitudinal panel-based monitoring (interpret longitudinally where possible) [8,21,23,95].
III. Systemic amplification	MetS; chronic low-grade inflammation; endothelial dysfunction; mitochondrial stress	AGEs–RAGE → NOX/ROS; inflammasome signaling (e.g., NLRP3 axis)	Sustained “silent inflammation”; worsened insulin resistance; vascular inflammation	Clinically established: hs-CRP/CRP, IL-6, CBC-derived indices (e.g., neutrophil-to-lymphocyte ratio). Research/exploratory: oxidative DNA/lipid damage markers (8-OHdG, F2-IsoPs), AGEs/sRAGE, mitochondrial DAMPs (mtDNA-derived). Therapeutic perspectives: cardiometabolic risk-factor management; redox-informed interpretation of inflammatory panels [17,28,58,174].
IV. Neurobiological “second hit” + feedback	Peripheral cytokines/redox mediators; BBB stress; neuroimmune activation	NOX4-linked microglial activation; pro-inflammatory polarization; oxidative injury	Potential contribution to cognitive decline/symptom burden; reinforcement of systemic–oral feedback loops	Clinically established: peripheral cytokine panels (IL-6, CRP) and longitudinal metabolic monitoring, already used in psychiatric care. Research/exploratory: integrated inflammation + redox panels with harmonized psychiatric and periodontal phenotyping; brain imaging/CSF correlates of neuroinflammation. Therapeutic perspectives: integrated psychiatry–dentistry–metabolic care pathways; phenotype-guided redox adjuncts (e.g., NAC, MitoQ) tested in stratified trials [11,17,146]. In parallel, mechanistic SCZ-facing evidence supports NLRP3 as a plausible neuroimmune node in an inflammatory subset, based on NLRP3-dependent astroglial inflammatory phenotypes in patient-derived iPSC models [173].

Abbreviations: 8-OHdG, 8-hydroxy-2′-deoxyguanosine; AGEs–RAGE, advanced glycation end-products and their receptors; AO, antioxidant; BBB, blood–brain barrier; BMI, body mass index; BOP, bleeding on probing; CAL, clinical attachment loss; CBC, complete blood count; F2-IsoPs, F2-isoprostanes; GCF, gingival crevicular fluid; HbA1c, glycated hemoglobin; hs-CRP, high-sensitivity C-reactive protein; IL, interleukin; MetS, metabolic syndrome; MP, matrix metalloproteinase; NLR, neutrophil-to-lymphocyte ratio; NOX, NADPH oxidase; PI3K/AKT/mTOR, phosphoinositide 3-kinase/protein kinase B/mechanistic target of rapamycin; RANKL/OPG, receptor activator of NF-κB ligand/osteoprotegerin; ROS/RNS, reactive oxygen/nitrogen species; SGAs, second-generation antipsychotics; TLR, toll-like receptor; TNF-α, tumor necrosis factor-alpha.

## Data Availability

No new primary datasets were generated in this study. All extracted evidence and search strategies supporting the claims in this paper are available within the article and its Supplementary Materials (Tables S1 and S2).

## Supplementary Materials

The following supporting information can be downloaded at: https://www.mdpi.com/article/10.3390/antiox15060679/s1, Table S1: Key systemic biomarker effects of non-surgical periodontal therapy (NSPT) cited in Section 1.3. Table S2: Targeted search strategy, screening approach, and selection guardrails (updated 8 March 2026).

## Abbreviations

The following abbreviations are used in this manuscript:

8-OHdG	8-hydroxy-2′-deoxyguanosine
ACVD	atherosclerotic cardiovascular disease
AGEs	advanced glycation end-products
AKT	protein kinase B
AO	antioxidant
ASC	apoptosis-associated speck-like protein containing a CARD
ATP	adenosine triphosphate
BBB	blood–brain barrier
BioAgeAccel	biological age acceleration
BMD	bone mineral density
BMI	body mass index
BOP	bleeding on probing
CAL	clinical attachment loss
CAT	catalase
CBC	complete blood count
CNFEST	Chinese First-Episode Schizophrenia Trial
CNS	central nervous system
COX6A2	cytochrome c oxidase subunit 6A2
CRP	C-reactive protein
DAMPs	damage-associated molecular patterns
DM	diabetes mellitus
esRAGE	endogenous secretory receptor for advanced glycation end-products
F2-IsoPs	F2-isoprostanes
GABA	γ-aminobutyric acid
GBR	GBR 12909
GCF	gingival crevicular fluid
*Gclm*-KO	glutamate-cysteine ligase modifier subunit knockout
GPx	glutathione peroxidase
GSH	reduced glutathione
HbA1c	glycated hemoglobin
HO-1	heme oxygenase-1
HPA	hypothalamic–pituitary–adrenal (axis)
hs-CRP	high-sensitivity C-reactive protein
IL	interleukin
IL-1β	interleukin 1β
IL-6	interleukin 6
IL-17	interleukin 17
IL-18	interleukin 18
KEAP1	Kelch-like ECH-associated protein 1
LBRI	low baseline BMI with rapid increase
LPS	lipopolysaccharide (s)
MDA	malondialdehyde
MetS	metabolic syndrome
Mfsd2a	major facilitated superfamily domain-containing 2A
*miR*-137	microRNA-137
MitoQ	mitoquinone mesylate
MMP	matrix metalloproteinase
mPTP	mitochondrial permeability transition pore
mtDNA	mitochondrial DNA
mtROS	mitochondrial reactive oxygen species
NAC	N-acetylcysteine
NADPH	nicotinamide adenine dinucleotide phosphate
NF-κB	nuclear factor-κ B
NHANES	National Health and Nutrition Examination Survey
NLRP3	NLR family pyrin domain-containing protein 3
NOX	NADPH oxidase
NOX2	NADPH oxidase 2
NOX4	NADPH oxidase 4
NRF2	nuclear factor erythroid 2-related factor 2
NSPT	non-surgical periodontal therapy
OPG	osteoprotegerin
OR	odds ratio
OXPHOS	oxidative phosphorylation
*P. gingivalis*	*Porphyromonas gingivalis*
PAMPs	pathogen-associated molecular patterns
PD	periodontitis (also referred to as periodontal disease)
PhenoAgeAccel	phenotypic age acceleration
PI3K/AKT/mTOR	phosphoinositide 3-kinase/protein kinase B/mammalian target of rapamycin
PMN	polymorphonuclear neutrophil
POST	personalized/precise oxidative stress therapy
PRISMA	Preferred Reporting Items for Systematic Reviews and Meta-Analyses
PRR	pattern recognition receptor
PVI	parvalbumin interneurons
PZ	psychosis
RAGE	receptor for advanced glycation end-products
RANKL	receptor activator of nuclear factor κ-B ligand
RCT	randomized controlled trial
RNS	reactive nitrogen species
ROS	reactive oxygen species
RyR	ryanodine receptor
SCs	respiratory chain supercomplexes
SCZ	schizophrenia
SERCA	sarco/endoplasmic reticulum Ca^2+^ ATPase
SES	socioeconomic status
SGAs	second-generation antipsychotics
SIRT1	sirtuin 1
SMI	severe mental illness
sNOX2-dp	soluble NOX2-derived peptide
SOD	superoxide dismutase
sRAGE	soluble receptor for advanced glycation end-products
SRP	scaling and root planing
SSD	schizophrenia-spectrum disorders
T2DM	type 2 diabetes mellitus
TAC	total antioxidant capacity
TLR	Toll-like receptor
TLR9	Toll-like receptor 9
TNF-α	tumor necrosis factor-alpha
TXNIP	thioredoxin-interacting protein
