# Supplementary material for: Silent Inflammation: A Critical Narrative Review of the Relationship Between Periodontal Disease and Psychosis—The Role of Oxidative Stress and Iatrogenic Comorbidities
Source: Antioxidants (Basel). 2026 May 28;15(6):679. doi: 10.3390/antiox15060679 (PMC13295320; doi:10.3390/antiox15060679)
Supplement: Supplementary file 1 [file antioxidants-15-00679-s001.zip › Supplementary Table S1.docx]

**Supplementary Table S1. Key systemic biomarker effects of non-surgical periodontal therapy (NSPT) cited in Section 1.3.**

| Study | Design | Population | Outcome | Effect size | Limitations |
| --- | --- | --- | --- | --- | --- |
| Luthra et al., 2023 (J Clin Periodontol) [53] | Systematic review + meta-analysis of RCTs (26 RCTs); follow-up ≥6 months | n=2579; periodontitis (mixed populations; incl. comorbidities) | Serum CRP after periodontal therapy | CRP reduced by 0.69 mg/L (95% CI −0.97 to −0.40) at 6 months; greatest reductions when baseline CRP >3 mg/L | High heterogeneity; limited evidence beyond 6 months; protocols/populations vary. https://pubmed.ncbi.nlm.nih.gov/35946825/ |
| Matsuda et al., 2024 (Am J Med) [56] | Multicenter observational study (STROBE); initial periodontal treatment | n=153; systemically healthy adults with periodontitis (22 centers, Japan) | Serum IL-6 and hsCRP after treatment | Direction: IL-6 and hsCRP decreased post-treatment. Reported medians: IL-6 (pg/mL) low responders 1.65→1.3; high responders 2.8→1.3. hsCRP low responders 1.96→1.64; high responders 2.06→1.18 | Non-randomized; variable follow-up (reported 50–690 days); responder stratification may introduce confounding/regression-to-mean. https://www.amjmed.com/article/S0002-9343(23)00706-4/fulltext |
| Sun et al., 2024 (J Dent) [36] | Systematic review + meta-analysis; mainly self-controlled case-series + 1 non-randomized interventional study | Periodontitis without diabetes; 22 studies; n=469 | HbA1c change after NSPT (non-diabetics) | At 3 months: 0.16% (95% CI 0.04–0.27; p=0.008). At 6 months: 0.17% (95% CI 0.08–0.27; p<0.001). (Modest absolute percentage-point change; interpret cautiously.) | Predominantly non-RCT/self-controlled; heterogeneity (smoking, sex, baseline HbA1c, prior therapy); causality limited. https://pubmed.ncbi.nlm.nih.gov/38642823/ |
| Bechina et al., 2025 (Oral Health Prev Dent) [54] | Systematic review (PRISMA) of NSPT and cardiovascular risk biomarkers | Adults with moderate–severe periodontitis; with/without comorbidities (16 included studies) | Systemic inflammation biomarkers (hsCRP, IL-6) after NSPT | Qualitative: NSPT associated with significant reduction in hsCRP and pro-inflammatory cytokines (incl. IL-6, TNF-α) | Abstract does not provide pooled numeric estimates; biomarker panels/assays and follow-up timepoints vary across included trials; heterogeneity across vascular endpoints. https://pubmed.ncbi.nlm.nih.gov/40842443/ |
| Simpson et al., 2022 (Cochrane Review; often cited in-text as “Simpson/Cochrane”) [55] | Systematic review of RCTs (Cochrane Database Syst Rev; CD004714.pub4) | People with diabetes mellitus (primarily type 2) + periodontitis; 35 RCTs; 3249 randomized | HbA1c after periodontal treatment (subgingival instrumentation) | Absolute HbA1c reduction: −0.43% at 3–4 months (95% CI −0.59 to −0.28; 30 studies, 2443 analysed); −0.30% at 6 months (95% CI −0.52 to −0.08; 12 studies, 1457); −0.50% at 12 months (95% CI −0.55 to −0.45; 1 study, 264) | Risk of bias: only 2 studies low risk; many high/unclear; adverse effects often not evaluated. https://pubmed.ncbi.nlm.nih.gov/35420698/ |

**Abbreviations**: CI, confidence interval; CRP, C-reactive protein; HbA1c, glycated hemoglobin; IL-6, interleukin 6; non-RCT, non-randomized controlled trial; NSPT, non-surgical periodontal therapy; PRISMA, Preferred Reporting Items for Systematic Reviews and Meta-Analyses; RCT, randomized controlled trial; STROBE, Strengthening the Reporting of Observational Studies in Epidemiology; TNF-α, tumor necrosis factor alpha.
