# Supplementary material for: Silent Inflammation: A Critical Narrative Review of the Relationship Between Periodontal Disease and Psychosis—The Role of Oxidative Stress and Iatrogenic Comorbidities
Source: Antioxidants (Basel). 2026 May 28;15(6):679. doi: 10.3390/antiox15060679 (PMC13295320; doi:10.3390/antiox15060679)
Supplement: Supplementary file 1 [file antioxidants-15-00679-s001.zip › Supplementary_Table_S2_FIXED.docx]

**Supplementary Table S2. Targeted search strategy, screening approach, and selection guardrails (updated 8 March 2026).**

| **Component** | **What was done** | **Rationale / how used in the manuscript** |
| --- | --- | --- |
| Databases | PubMed/MEDLINE (primary); Google Scholar (complementary). | PubMed for biomedical traceability (PMID/DOI); Scholar for interdisciplinary/early-online items. |
| Search strings (PubMed) | **Block A (periodontal exposure × psychosis/SMI):** ("periodontitis"[MeSH] OR "periodontal disease"[tiab] OR "tooth loss"[tiab]) AND ("psychosis"[MeSH] OR "schizophrenia"[MeSH] OR "schizophrenia spectrum"[tiab] OR "severe mental illness"[tiab] OR "psychotic disorders"[tiab]).  **Block B (periodontal exposure × oxidative stress / inflammasome / mechanisms):** ("periodontitis"[MeSH] OR "periodontal"[tiab]) AND ("oxidative stress"[MeSH] OR "TXNIP"[tiab] OR "NLRP3"[tiab] OR "inflammasome"[tiab] OR "advanced glycation end products"[MeSH] OR "RAGE receptor"[tiab] OR "NADPH oxidase"[MeSH] OR "blood-brain barrier"[MeSH] OR "neuroinflammation"[tiab] OR "mitochondrial DAMPs"[tiab]).  **Filters applied:** English; date 2000–8 March 2026; humans/animals as appropriate per claim. Block A and Block B were intersected when needed; key references identified via backward and forward citation tracking from anchor papers. | Enables reproducibility and auditability. |
| Timepoint | Last updated: 8 March 2026. | Fixes the “cut-off” for what is considered “current” evidence. |
| Inclusion (clinical effects) | Systematic reviews/meta-analyses and RCTs prioritized for NSPT → systemic biomarkers. Human studies prioritized for clinical-effect claims. | Prevents over-reliance on lower-quality designs for effect-size claims. |
| Inclusion (mechanisms) | Mechanistic/translational studies included when clinical evidence is limited; preference for reviews and well-characterized experimental models. | Supports plausibility claims without overstating causality. |
| Exclusion rules | Off-topic (no periodontal exposure / no psychosis/SMI relevance), no interpretable biomarker outcomes for the specific claim, duplicates/overlaps without added value, irretrievable full text when details were needed to support a statement. | Clarifies boundaries and reduces selection ambiguity. |
| Screening workflow | Title/abstract screening → full-text check for key items; emphasis on highly cited and/or recent studies; record-level verification in PubMed when possible. | Explains how “targeted” screening was implemented. |
| Verification | DOI/PMID cross-check in PubMed; Crossref used to resolve bibliographic discrepancies (author order, year, journal fields). | Ensures reference integrity and reduces citation errors. |
| Citation tracking | Backward citation tracking from key papers; optionally forward tracking for seminal items (if performed). | Captures influential papers that keyword search may miss. |
| Evidence summary linkage | Key clinical biomarker-effect statements linked to Supplementary Table S1 (effect sizes + limitations). | Direct bridge between claims and underlying evidence. |

**Abbreviations**: DOI, Digital Object Identifier; MEDLINE, Medical Literature Analysis and Retrieval System Online; NSPT, non-surgical periodontal therapy; PMID, PubMed identifier; RCT, randomized controlled trial; SMI, severe mental illness.
